# ISARIC-COVID-19 dataset: A Prospective, Standardized, Global Dataset of Patients Hospitalized with COVID-19

**DOI:** 10.1038/s41597-022-01534-9

**Published:** 2022-07-30

**Authors:** ISARIC Clinical Characterization Group, Ali Abbas, Sheryl Ann Abdukahil, Nurul Najmee Abdulkadir, Ryuzo Abe, Laurent Abel, Lara Absil, Kamal Abu Jabal, Hiba Abu Zayyad, Subhash Acharya, Andrew Acker, Shingo Adachi, Elisabeth Adam, Enrico Adriano, Diana Adrião, Saleh Al Ageel, Shakeel Ahmed, Marina Aiello, Kate Ainscough, Eka Airlangga, Tharwat Aisa, Ali Ait Hssain, Younes Ait Tamlihat, Takako Akimoto, Ernita Akmal, Eman Al Qasim, Tala Al-dabbous, Abdulrahman Al-Fares, Razi Alalqam, Angela Alberti, Senthilkumar Alegesan, Cynthia Alegre, Marta Alessi, Beatrice Alex, Kévin Alexandre, Huda Alfoudri, Adam Ali, Imran Ali, Naseem Ali Shah, Naseem Ali Sheikh, Kazali Enagnon Alidjnou, Jeffrey Aliudin, Qabas Alkhafajee, Clotilde Allavena, Nathalie Allou, Aneela Altaf, João Alves, João Melo Alves, Rita Alves, Maria Amaral, Nur Amira, Heidi Ammerlaan, Phoebe Ampaw, Roberto Andini, Claire Andrejak, Andrea Angheben, François Angoulvant, Séverine Ansart, Sivanesen Anthonidass, Massimo Antonelli, Carlos Alexandre Antunes de Brito, Kazi Rubayet Anwar, Ardiyan Apriyana, Yaseen Arabi, Irene Aragao, Francisco Arancibia, Carolline Araujo, Antonio Arcadipane, Patrick Archambault, Lukas Arenz, Jean-Benoît Arlet, Christel Arnold-Day, Ana Aroca, Lovkesh Arora, Rakesh Arora, Elise Artaud-Macari, Diptesh Aryal, Motohiro Asaki, Angel Asensio, Elizabeth Ashley, Muhammad Ashraf, Namra Asif, Mohammad Asim, Jean Baptiste Assie, Amirul Asyraf, Anika Atique, A. M. Udara Lakshan Attanyake, Johann Auchabie, Hugues Aumaitre, Adrien Auvet, Eyvind W. Axelsen, Laurène Azemar, Cecile Azoulay, Benjamin Bach, Delphine Bachelet, Claudine Badr, Roar Bævre-Jensen, Nadia Baig, J. Kenneth Baillie, J Kevin Baird, Erica Bak, Agamemnon Bakakos, Nazreen Abu Bakar, Andriy Bal, Mohanaprasanth Balakrishnan, Valeria Balan, Firouzé Bani-Sadr, Renata Barbalho, Nicholas Yuri Barbosa, Wendy S. Barclay, Saef Umar Barnett, Michaela Barnikel, Helena Barrasa, Audrey Barrelet, Cleide Barrigoto, Marie Bartoli, Cheryl Bartone, Joaquín Baruch, Mustehan Bashir, Romain Basmaci, Muhammad Fadhli Hassin Basri, Denise Battaglini, Jules Bauer, Diego Fernando Bautista Rincon, Denisse Bazan Dow, Abigail Beane, Alexandra Bedossa, Ker Hong Bee, Husna Begum, Sylvie Behilill, Karine Beiruti, Albertus Beishuizen, Aleksandr Beljantsev, David Bellemare, Anna Beltrame, Beatriz Amorim Beltrão, Marine Beluze, Nicolas Benech, Lionel Eric Benjiman, Dehbia Benkerrou, Suzanne Bennett, Luís Bento, Jan-Erik Berdal, Delphine Bergeaud, Hazel Bergin, José Luis Bernal Sobrino, Giulia Bertoli, Lorenzo Bertolino, Simon Bessis, Adam Betz, Sybille Bevilcaqua, Karine Bezulier, Amar Bhatt, Krishna Bhavsar, Isabella Bianchi, Claudia Bianco, Farah Nadiah Bidin, Moirangthem Bikram Singh, Felwa Bin Humaid, Mohd Nazlin Bin Kamarudin, François Bissuel, Patrick Biston, Laurent Bitker, Jonathan Bitton, Pablo Blanco-Schweizer, Catherine Blier, Frank Bloos, Mathieu Blot, Lucille Blumberg, Filomena Boccia, Laetitia Bodenes, Alice Bogaarts, Debby Bogaert, Anne-Hélène Boivin, Pierre-Adrien Bolze, François Bompart, Patrizia Bonelli, Aurelius Bonfasius, Diogo Borges, Raphaël Borie, Hans Martin Bosse, Elisabeth Botelho-Nevers, Lila Bouadma, Olivier Bouchaud, Sabelline Bouchez, Dounia Bouhmani, Damien Bouhour, Kévin Bouiller, Laurence Bouillet, Camile Bouisse, Thipsavanh Bounphiengsy, Latsaniphone Bountthasavong, Anne-Sophie Boureau, John Bourke, Maude Bouscambert, Aurore Bousquet, Jason Bouziotis, Bianca Boxma, Marielle Boyer-Besseyre, Maria Boylan, Fernando Augusto Bozza, Axelle Braconnier, Cynthia Braga, Timo Brandenburger, Filipa Brás Monteiro, Luca Brazzi, Dorothy Breen, Patrick Breen, Kathy Brickell, Alex Browne, Shaunagh Browne, Nicolas Brozzi, Sonja Hjellegjerde Brunvoll, Marjolein Brusse-Keizer, Nina Buchtele, Christian Buesaquillo, Polina Bugaeva, Marielle Buisson, Danilo Buonsenso, Erlina Burhan, Aidan Burrell, Ingrid G. Bustos, Denis Butnaru, André Cabie, Susana Cabral, Eder Caceres, Cyril Cadoz, Mia Callahan, Kate Calligy, Jose Andres Calvache, Caterina Caminiti, João Camões, Valentine Campana, Paul Campbell, Josie Campisi, Cecilia Canepa, Mireia Cantero, Pauline Caraux-Paz, Sheila Cárcel, Chiara Simona Cardellino, Filipa Cardoso, Filipe Cardoso, Nelson Cardoso, Sofia Cardoso, Simone Carelli, Francesca Carlacci, Nicolas Carlier, Thierry Carmoi, Gayle Carney, Inês Carqueja, Marie-Christine Carret, François Martin Carrier, Ida Carroll, Gail Carson, Leonor Carvalho, Maire-Laure Casanova, Mariana Cascão, Siobhan Casey, José Casimiro, Bailey Cassandra, Silvia Castañeda, Nidyanara Castanheira, Guylaine Castor-Alexandre, Henry Castrillón, Ivo Castro, Ana Catarino, François-Xavier Catherine, Paolo Cattaneo, Roberta Cavalin, Giulio Giovanni Cavalli, Alexandros Cavayas, Adrian Ceccato, Minerva Cervantes-Gonzalez, Anissa Chair, Catherine Chakveatze, Adrienne Chan, Meera Chand, Christelle Chantalat Auger, Jean-Marc Chapplain, Julie Chas, Allegra Chatterjee, Mobin Chaudry, Jonathan Samuel Chávez Iñiguez, Anjellica Chen, Yih-Sharng Chen, Matthew Pellan Cheng, Antoine Cheret, Alfredo Antonio Chetta, Thibault Chiarabini, Julian Chica, Suresh Kumar Chidambaram, Leong Chin Tho, Catherine Chirouze, Davide Chiumello, Hwa Jin Cho, Sung-Min Cho, Bernard Cholley, Danoy Chommanam, Marie-Charlotte Chopin, Ting Soo Chow, Yock Ping Chow, Nathaniel Christy, Hiu Jian Chua, Jonathan Chua, Jose Pedro Cidade, José Miguel Cisneros Herreros, Barbara Wanjiru Citarella, Anna Ciullo, Emma Clarke, Jennifer Clarke, Rolando Claure-Del Granado, Sara Clohisey, Perren J. Cobb, Cassidy Codan, Caitriona Cody, Alexandra Coelho, Megan Coles, Gwenhaël Colin, Michael Collins, Sebastiano Maria Colombo, Pamela Combs, Marie Connor, Anne Conrad, Sofía Contreras, Elaine Conway, Graham S. Cooke, Mary Copland, Hugues Cordel, Amanda Corley, Sabine Cornelis, Alexander Daniel Cornet, Arianne Joy Corpuz, Andrea Cortegiani, Grégory Corvaisier, Emma Costigan, Camille Couffignal, Sandrine Couffin-Cadiergues, Roxane Courtois, Stéphanie Cousse, Rachel Cregan, Charles Crepy D’Orleans, Sabine Croonen, Gloria Crowl, Jonathan Crump, Claudina Cruz, Juan Luis Cruz Berm, Jaime Cruz Rojo, Marc Csete, Alberto Cucino, Ailbhe Cullen, Matthew Cummings, Ger Curley, Gerard Curley, Elodie Curlier, Colleen Curran, Paula Custodio, Federico D’Amico, Frédérick D’Aragon, Eric D’Ortenzio, Ana da Silva Filipe, Charlene Da Silveira, Al-Awwab Dabaliz, Andrew Dagens, John Arne Dahl, Darren Dahly, Heidi Dalton, Jo Dalton, Seamus Daly, Nick Daneman, Corinne Daniel, Emmanuelle A Dankwa, Jorge Dantas, Mark de Boer, Gillian de Loughry, Diego de Mendoza, Etienne De Montmollin, Rafael Freitas de Oliveira França, Ana Isabel de Pinho Oliveira, Rosanna De Rosa, Cristina De Rose, Thushan de Silva, Peter de Vries, Jillian Deacon, David Dean, Alexa Debard, Bianca DeBenedictis, Marie-Pierre Debray, Nathalie DeCastro, William Dechert, Lauren Deconninck, Romain Decours, Eve Defous, Isabelle Delacroix, Eric Delaveuve, Karen Delavigne, Nathalie M. Delfos, Ionna Deligiannis, Andrea Dell’Amore, Christelle Delmas, Pierre Delobel, Corine Delsing, Elisa Demonchy, Emmanuelle Denis, Dominique Deplanque, Pieter Depuydt, Mehul Desai, Diane Descamps, Mathilde Desvallées, Santi Dewayanti, Pathik Dhanger, Alpha Diallo, Sylvain Diamantis, André Dias, Andrea Dias, Juan Jose Diaz, Priscila Diaz, Rodrigo Diaz, Kévin Didier, Jean-Luc Diehl, Wim Dieperink, Jérôme Dimet, Vincent Dinot, Fara Diop, Alphonsine Diouf, Yael Dishon, Félix Djossou, Annemarie B. Docherty, Helen Doherty, Arjen M Dondorp, Andy Dong, Christl A. Donnelly, Maria Donnelly, Chloe Donohue, Sean Donohue, Yoann Donohue, Peter Doran, Céline Dorival, Phouvieng Douangdala, James Joshua Douglas, Renee Douma, Nathalie Dournon, Triona Downer, Joanne Downey, Mark Downing, Tom Drake, Aoife Driscoll, Amiel A. Dror, Murray Dryden, Claudio Duarte Fonseca, Vincent Dubee, François Dubos, Audrey Dubot-Pérès, Alexandre Ducancelle, Toni Duculan, Susanne Dudman, Abhijit Duggal, Paul Dunand, Jake Dunning, Mathilde Duplaix, Emanuele Durante-Mangoni, Lucian Durham, Bertrand Dussol, Juliette Duthoit, Xavier Duval, Anne Margarita Dyrhol-Riise, Sim Choon Ean, Marco Echeverria-Villalobos, Giorgio Economopoulos, Michael Edelstein, Siobhan Egan, Linn Margrete Eggesbø, Carla Eira, Mohammed El Sanharawi, Subbarao Elapavaluru, Brigitte Elharrar, Jacobien Ellerbroek, Merete Ellingjord-Dale, Philippine Eloy, Tarek Elshazly, Iqbal Elyazar, Isabelle Enderle, Tomoyuki Endo, Chan Chee Eng, Ilka Engelmann, Vincent Enouf, Olivier Epaulard, Martina Escher, Mariano Esperatti, Hélène Esperou, Marina Esposito-Farese, João Estevão, Manuel Etienne, Nadia Ettalhaoui, Anna Greti Everding, Mirjam Evers, Isabelle Fabre, Marc Fabre, Amna Faheem, Arabella Fahy, Cameron J. Fairfield, Zul Fakar, Komal Fareed, Pedro Faria, Ahmed Farooq, Hanan Fateena, Arie Zainul Fatoni, Karine Faure, Raphaël Favory, Mohamed Fayed, Niamh Feely, Laura Feeney, Jorge Fernandes, Marília Andreia Fernandes, Susana Fernandes, François-Xavier Ferrand, Eglantine Ferrand Devouge, Joana Ferrão, Carlo Ferrari, Mário Ferraz, Benigno Ferreira, Isabel Ferreira, Sílvia Ferreira, Ricard Ferrer-Roca, Nicolas Ferriere, Céline Ficko, Claudia Figueiredo-Mello, William Finlayson, Juan Fiorda, Thomas Flament, Clara Flateau, Tom Fletcher, Letizia Lucia Florio, Brigid Flynn, Deirdre Flynn, Federica Fogliazza, Claire Foley, Jean Foley, Victor Fomin, Tatiana Fonseca, Patricia Fontela, Simon Forsyth, Denise Foster, Giuseppe Foti, Erwan Fourn, Robert A. Fowler, Marianne Fraher, Diego Franch-Llasat, Christophe Fraser, John F Fraser, Marcela Vieira Freire, Ana Freitas Ribeiro, Craig French, Caren Friedrich, Ricardo Fritz, Stéphanie Fry, Nora Fuentes, Masahiro Fukuda, G Argin, Valérie Gaborieau, Rostane Gaci, Massimo Gagliardi, Jean-Charles Gagnard, Nathalie Gagné, Amandine Gagneux-Brunon, Sérgio Gaião, Linda Gail Skeie, Phil Gallagher, Elena Gallego Curto, Carrol Gamble, Yasmin Gani, Arthur Garan, Rebekha Garcia, Noelia García Barrio, Julia Garcia-Diaz, Esteban Garcia-Gallo, Navya Garimella, Federica Garofalo, Denis Garot, Valérie Garrait, Basanta Gauli, Nathalie Gault, Aisling Gavin, Anatoliy Gavrylov, Alexandre Gaymard, Johannes Gebauer, Eva Geraud, Louis Gerbaud Morlaes, Nuno Germano, Praveen Kumar Ghisulal, Jade Ghosn, Marco Giani, Carlo Giaquinto, Jess Gibson, Tristan Gigante, Morgane Gilg, Elaine Gilroy, Guillermo Giordano, Michelle Girvan, Valérie Gissot, Jesse Gitaka, Gezy Giwangkancana, Daniel Glikman, Petr Glybochko, Eric Gnall, Geraldine Goco, François Goehringer, Siri Goepel, Jean-Christophe Goffard, Jin Yi Goh, Jonathan Golob, Rui Gomes, Kyle Gomez, Joan Gómez-Junyent, Marie Gominet, Bronner P. Gonçalves, Alicia Gonzalez, Patricia Gordon, Yanay Gorelik, Isabelle Gorenne, Laure Goubert, Cécile Goujard, Tiphaine Goulenok, Margarite Grable, Jeronimo Graf, Edward Wilson Grandin, Pascal Granier, Giacomo Grasselli, Lorenzo Grazioli, Christopher A. Green, Courtney Greene, William Greenhalf, Segolène Greffe, Domenico Luca Grieco, Matthew Griffee, Fiona Griffiths, Ioana Grigoras, Albert Groenendijk, Anja Grosse Lordemann, Heidi Gruner, Yusing Gu, Fabio Guarracino, Jérémie Guedj, Martin Guego, Dewi Guellec, Anne-Marie Guerguerian, Daniela Guerreiro, Romain Guery, Anne Guillaumot, Laurent Guilleminault, Maisa Guimarães de Castro, Thomas Guimard, Marieke Haalboom, Daniel Haber, Hannah Habraken, Ali Hachemi, Amy Hackmann, Nadir Hadri, Fakhir Haidri, Sheeba Hakak, Adam Hall, Matthew Hall, Sophie Halpin, Jawad Hameed, Ansley Hamer, Raph L. Hamers, Rebecca Hamidfar, Bato Hammarström, Terese Hammond, Lim Yuen Han, Rashan Haniffa, Kok Wei Hao, Hayley Hardwick, Ewen M. Harrison, Janet Harrison, Samuel Bernard Ekow Harrison, Alan Hartman, Mohd Shahnaz Hasan, Junaid Hashmi, Madiha Hashmi, Ailbhe Hayes, Leanne Hays, Jan Heerman, Lars Heggelund, Ross Hendry, Martina Hennessy, Aquiles Henriquez-Trujillo, Maxime Hentzien, Jaime Hernandez-Montfort, Daniel Herr, Andrew Hershey, Liv Hesstvedt, Astarini Hidayah, Dawn Higgins, Eibhilin Higgins, Rita Hinchion, Samuel Hinton, Hiroaki Hiraiwa, Haider Hirkani, Hikombo Hitoto, Antonia Ho, Yi Bin Ho, Alexandre Hoctin, Isabelle Hoffmann, Wei Han Hoh, Oscar Hoiting, Rebecca Holt, Jan Cato Holter, Peter Horby, Juan Pablo Horcajada, Koji Hoshino, Kota Hoshino, Ikram Houas, Catherine L. Hough, Stuart Houltham, Jimmy Ming-Yang Hsu, Jean-Sébastien Hulot, Stella Huo, Iqbal Hussain, Samreen Ijaz, Hajnal-Gabriela Illes, Patrick Imbert, Mohammad Imran, Rana Imran Sikander, Aftab Imtiaz, Hugo Inácio, Carmen Infante Dominguez, Yun Sii Ing, Elias Iosifidis, Mariachiara Ippolito, Sarah Isgett, Tiago Isidoro, Nadiah Ismail, Margaux Isnard, Mette Stausland Istre, Junji Itai, Asami Ito, Daniel Ivulich, Danielle Jaafar, Salma Jaafoura, Julien Jabot, Clare Jackson, Nina Jamieson, Pierre Jaquet, Waasila Jassat, Coline Jaud-Fischer, Stéphane Jaureguiberry, Jeffrey Javidfar, Denise Jaworsky, Florence Jego, Anilawati Mat Jelani, Synne Jenum, Ruth Jimbo-Sotomayor, Ong Yiaw Joe, Ruth N. Jorge García, Silje Bakken Jørgensen, Cédric Joseph, Mark Joseph, Swosti Joshi, Mercé Jourdain, Philippe Jouvet, Jennifer June, Anna Jung, Hanna Jung, Dafsah Juzar, Ouifiya Kafif, Florentia Kaguelidou, Neerusha Kaisbain, Thavamany Kaleesvran, Sabina Kali, Alina Kalicinska, Karl Trygve Kalleberg, Smaragdi Kalomoiri, Muhammad Aisar Ayadi Kamaluddin, Zul Amali Che Kamaruddin, Nadiah Kamarudin, Kavita Kamineni, Darshana Hewa Kandamby, Chris Kandel, Kong Yeow Kang, Darakhshan Kanwal, Dyah Kanyawati, Pratap Karpayah, Todd Karsies, Christiana Kartsonaki, Daisuke Kasugai, Anant Kataria, Kevin Katz, Aasmine Kaur, Tatsuya Kawasaki, Christy Kay, Hannah Keane, Seán Keating, Pulak Kedia, Andrea Kelly, Aoife Kelly, Claire Kelly, Niamh Kelly, Sadie Kelly, Yvelynne Kelly, Maeve Kelsey, Ryan Kennedy, Kalynn Kennon, Sommay Keomany, Maeve Kernan, Younes Kerroumi, Sharma Keshav, Evelyne Kestelyn, Imrana Khalid, Osama Khalid, Antoine Khalil, Coralie Khan, Irfan Khan, Quratul Ain Khan, Sushil Khanal, Abid Khatak, Amin Khawaja, Krish Kherajani, Michelle E. Kho, Denisa Khoo, Ryan Khoo, Saye Khoo, Nasir Khoso, Khor How Kiat, Yuri Kida, Harrison Kihuga, Peter Kiiza, Beathe Kiland Granerud, Anders Benjamin Kildal, Jae Burm Kim, Antoine Kimmoun, Detlef Kindgen-Milles, Alexander King, Nobuya Kitamura, Eyrun Floerecke Kjetland Kjetland, Paul Klenerman, Rob Klont, Gry Kloumann Bekken, Stephen R Knight, Robin Kobbe, Chamira Kodippily, Malte Kohns Vasconcelos, Sabin Koirala, Mamoru Komatsu, Volkan Korten, Caroline Kosgei, Arsène Kpangon, Karolina Krawczyk, Sudhir Krishnan, Vinothini Krishnan, Oksana Kruglova, Deepali Kumar, Ganesh Kumar, Mukesh Kumar, Pavan Kumar Vecham, Dinesh Kuriakose, Ethan Kurtzman, Neurinda Permata Kusumastuti, Demetrios Kutsogiannis, Galyna Kutsyna, Konstantinos Kyriakoulis, Erwan L’Her, Marie Lachatre, Marie Lacoste, John G. Laffey, Marie Lagrange, Fabrice Laine, Olivier Lairez, Sanjay Lakhey, Antonio Lalueza, Marc Lambert, François Lamontagne, Marie Langelot-Richard, Vincent Langlois, Eka Yudha Lantang, Marina Lanza, Cédric Laouénan, Samira Laribi, Delphine Lariviere, Stéphane Lasry, Sakshi Lath, Naveed Latif, Odile Launay, Didier Laureillard, Yoan Lavie-Badie, Andrew Law, Andy Law, Cassie Lawrence, Teresa Lawrence, Minh Le, Clément Le Bihan, Cyril Le Bris, Georges Le Falher, Lucie Le Fevre, Quentin Le Hingrat, Marion Le Maréchal, Soizic Le Mestre, Gwenaël Le Moal, Vincent Le Moing, Hervé Le Nagard, Paul Le Turnier, Ema Leal, Marta Leal Santos, Biing Horng Lee, Heng Gee Lee, James Lee, Su Hwan Lee, Todd C. Lee, Yi Lin Lee, Gary Leeming, Bénédicte Lefebvre, Laurent Lefebvre, Benjamin Lefevre, Sylvie LeGac, Jean-Daniel Lelievre, François Lellouche, Adrien Lemaignen, Véronique Lemee, Anthony Lemeur, Gretchen Lemmink, Ha Sha Lene, Jenny Lennon, Rafael León, Marc Leone, Michela Leone, Quentin Lepiller, François-Xavier Lescure, Olivier Lesens, Mathieu Lesouhaitier, Amy Lester-Grant, Andrew Letizia, Bruno Levy, Yves Levy, Claire Levy-Marchal, Katarzyna Lewandowska, Gianluigi Li Bassi, Janet Liang, Ali Liaquat, Geoffrey Liegeon, Kah Chuan Lim, Wei Shen Lim, Chantre Lima, Bruno Lina, Lim Lina, Andreas Lind, Maja Katherine Lingad, Guillaume Lingas, Sylvie Lion-Daolio, Samantha Lissauer, Keibun Liu, Marine Livrozet, Patricia Lizotte, Antonio Loforte, Navy Lolong, Leong Chee Loon, Diogo Lopes, Dalia Lopez-Colon, Anthony L. Loschner, Paul Loubet, Bouchra Loufti, Guillame Louis, Silvia Lourenco, Lara Lovelace-Macon, Lee Lee Low, Marije Lowik, Jia Shyi Loy, Jean Christophe Lucet, Carlos Lumbreras Bermejo, Carlos M. Luna, Olguta Lungu, Liem Luong, Nestor Luque, Dominique Luton, Nilar Lwin, Ruth Lyons, Olavi Maasikas, Oryane Mabiala, Sarah MacDonald, Moïse Machado, Gabriel Macheda, Juan Macias Sanchez, Jai Madhok, Hashmi Madiha, Guillermo Maestro de la Calle, Jacob Magara, Giuseppe Maglietta, Rafael Mahieu, Sophie Mahy, Ana Raquel Maia, Lars S. Maier, Mylène Maillet, Thomas Maitre, Maria Majori, Maximilian Malfertheiner, Nadia Malik, Paddy Mallon, Fernando Maltez, Denis Malvy, Patrizia Mammi, Victoria Manda, Jose M. Mandei, Laurent Mandelbrot, Frank Manetta, Julie Mankikian, Edmund Manning, Aldric Manuel, Ceila Maria Sant’Ana Malaque, Daniel Marino, Flávio Marino, Samuel Markowicz, Charbel Maroun Eid, Ana Marques, Catherine Marquis, Brian Marsh, Laura Marsh, Megan Marshal, John Marshall, Celina Turchi Martelli, Dori-Ann Martin, Emily Martin, Guillaume Martin-Blondel, Ignacio Martin-Loeches, Alejandro Martin-Quiros, Alessandra Martinelli, Martin Martinot, Ana Martins, João Martins, Nuno Martins, Caroline Martins Rego, Gennaro Martucci, Olga Martynenko, Eva Miranda Marwali, Marsilla Marzukie, Juan Fernado Masa Jimenez, David Maslove, Phillip Mason, Sabina Mason, Sobia Masood, Basri Mat Nor, Moshe Matan, Henrique Mateus Fernandes, Meghena Mathew, Daniel Mathieu, Mathieu Mattei, Romans Matulevics, Laurence Maulin, Michael Maxwell, Javier Maynar, Mayfong Mayxay, Thierry Mazzoni, Natalie Mc Evoy, Lisa Mc Sweeney, Colin McArthur, Aine McCarthy, Anne McCarthy, Colin McCloskey, Rachael McConnochie, Sherry McDermott, Sarah E. McDonald, Aine McElroy, Samuel McElwee, Victoria McEneany, Allison McGeer, Chris McKay, Johnny McKeown, Kenneth A. McLean, Paul McNally, Bairbre McNicholas, Elaine McPartlan, Edel Meaney, Cécile Mear-Passard, Maggie Mechlin, Maqsood Meher, Omar Mehkri, Ferruccio Mele, Luis Melo, Kashif Memon, Joao Mendes, Ogechukwu Menkiti, Kusum Menon, France Mentré, Alexander J. Mentzer, Emmanuelle Mercier, Noémie Mercier, Antoine Merckx, Mayka Mergeay-Fabre, Blake Mergler, Laura Merson, Tiziana Meschi, António Mesquita, Roberta Meta, Osama Metwally, Agnès Meybeck, Dan Meyer, Alison M. Meynert, Vanina Meysonnier, Amina Meziane, Mehdi Mezidi, Giuliano Michelagnoli, Céline Michelanglei, Isabelle Michelet, Efstathia Mihelis, Vladislav Mihnovit, Hugo Miranda-Maldonado, Nor Arisah Misnan, Nik Nur Eliza Mohamed, Tahira Jamal Mohamed, Asma Moin, David Molina, Elena Molinos, Brenda Molloy, Mary Mone, Agostinho Monteiro, Claudia Montes, Giorgia Montrucchio, Sarah Moore, Shona C. Moore, Lina Morales Cely, Lucia Moro, Diego Rolando Morocho Tutillo, Ben Morton, Catherine Motherway, Ana Motos, Hugo Mouquet, Clara Mouton Perrot, Julien Moyet, Caroline Mudara, Aisha Kalsoom Mufti, Ng Yong Muh, Dzawani Muhamad, Jimmy Mullaert, Fredrik Müller, Karl Erik Müller, Daniel Munblit, Syed Muneeb, Nadeem Munir, Laveena Munshi, Aisling Murphy, Lorna Murphy, Marlène Murris, Srinivas Murthy, Himed Musaab, Carlotta Mutti, Himasha Muvindi, Gugapriyaa Muyandy, Dimitra Melia Myrodia, Farah Nadia Mohd-Hanafiah, Dave Nagpal, Mangala Narasimhan, Nageswaran Narayanan, Rashid Nasim Khan, Alasdair Nazerali-Maitland, Nadège Neant, Holger Neb, Coca Necsoi, Nikita A. Nekliudov, Erni Nelwan, Raul Neto, Emily Neumann, Bernardo Neves, Pauline Yeung Ng, Wing Yiu Ng, Anthony Nghi, Jane Ngure, Duc Nguyen, Orna Ni Choileain, Niamh Ni Leathlobhair, Alistair Nichol, Prompak Nitayavardhana, Stephanie Nonas, Nurul Amani Mohd Noordin, Marion Noret, Nurul Faten Izzati Norharizam, Lisa Norman, Alessandra Notari, Mahdad Noursadeghi, Karolina Nowicka, Adam Nowinski, Saad Nseir, Jose I. Nunez, Nurnaningsih Nurnaningsih, Dwi Utomo Nusantara, Elsa Nyamankolly, Anders Benteson Nygaard, Fionnuala O. Brien, Annmarie O. Callaghan, Annmarie O’Callaghan, Max O’Donnell, Sophie O’Halloran, Katie O’Hearn, Conar O’Neil, Linda O’Shea, Miriam O’Sullivan, Giovanna Occhipinti, Derbrenn OConnor, Tawnya Ogston, Takayuki Ogura, Tak-Hyuk Oh, Shinichiro Ohshimo, Agnieszka Oldakowska, João Oliveira, Larissa Oliveira, Piero L. Olliaro, David S. Y. Ong, Jee Yan Ong, Wilna Oosthuyzen, Anne Opavsky, Peter Openshaw, Saijad Orakzai, Claudia Milena Orozco-Chamorro, Andrés Orquera, Jamel Ortoleva, Javier Osatnik, Siti Zubaidah Othman, Paul Otiku, Nadia Ouamara, Rachida Ouissa, Clark Owyang, Eric Oziol, Maïder Pagadoy, Justine Pages, Amanda Palacios, Mario Palacios, Massimo Palmarini, Giovanna Panarello, Prasan Kumar Panda, Hem Paneru, Lai Hui Pang, Mauro Panigada, Nathalie Pansu, Aurélie Papadopoulos, Paolo Parducci, Edwin Fernando Paredes Oña, Rachael Parke, Melissa Parker, Briseida Parra, Vieri Parrini, Taha Pasha, Jérémie Pasquier, Bruno Pastene, Fabian Patauner, Drashti Patel, Mohan Dass Pathmanathan, Luís Patrão, Patricia Patricio, Juliette Patrier, Laura Patrizi, Lisa Patterson, Rajyabardhan Pattnaik, Christelle Paul, Mical Paul, Jorge Paulos, William A. Paxton, Jean-François Payen, Kalaiarasu Peariasamy, Miguel Pedrera Jiménez, Giles J. Peek, Florent Peelman, Nathan Peiffer-Smadja, Vincent Peigne, Mare Pejkovska, Paolo Pelosi, Ithan D. Peltan, Rui Pereira, Daniel Perez, Luis Periel, Thomas Perpoint, Antonio Pesenti, Vincent Pestre, Lenka Petrou, Ventzislava Petrov-Sanchez, Michele Petrovic, Frank Olav Pettersen, Gilles Peytavin, Scott Pharand, Ooyanong Phonemixay, Michael Piagnerelli, Walter Picard, Olivier Picone, Maria de Piero, Carola Pierobon, Djura Piersma, Carlos Pimentel, Raquel Pinto, Catarina Pires, Isabelle Pironneau, Lionel Piroth, Roberta Pisi, Ayodhia Pitaloka, Riinu Pius, Simone Piva, Laurent Plantier, Hon Shen Png, Julien Poissy, Ryadh Pokeerbux, Maria Pokorska-Spiewak, Sergio Poli, Georgios Pollakis, Diane Ponscarme, Jolanta Popielska, Diego Bastos Porto, Andra-Maris Post, Douwe F. Postma, Pedro Povoa, Diana Póvoas, Jeff Powis, Sofia Prapa, Viladeth Praphasiri, Sébastien Preau, Christian Prebensen, Jean-Charles Preiser, Anton Prinssen, Mark G. Pritchard, Gamage Dona Dilanthi Priyadarshani, Lucia Proença, Sravya Pudota, Oriane Puéchal, Bambang Pujo Semedi, Mathew Pulicken, Matteo Puntoni, Gregory Purcell, Luisa Quesada, Vilmaris Quinones-Cardona, Víctor Quirós González, Else Quist-Paulsen, Mohammed Quraishi, Maia Rabaa, Christian Rabaud, Ebenezer Rabindrarajan, Aldo Rafael, Marie Rafiq, Gabrielle Ragazzo, Mutia Rahardjani, Ahmad Kashfi Haji Ab Rahman, Rozanah Abd Rahman, Arsalan Rahutullah, Fernando Rainieri, Giri Shan Rajahram, Pratheema Ramachandran, Nagarajan Ramakrishnan, Kollengode Ramanathan, Ahmad Afiq Ramli, Blandine Rammaert, Grazielle Viana Ramos, Anais Rampello, Asim Rana, Rajavardhan Rangappa, Ritika Ranjan, Elena Ranza, Christophe Rapp, Aasiyah Rashan, Thalha Rashan, Ghulam Rasheed, Menaldi Rasmin, Indrek Rätsep, Cornelius Rau, Francesco Rausa, Tharmini Ravi, Ali Raza, Andre Real, Stanislas Rebaudet, Sarah Redl, Brenda Reeve, Attaur Rehman, Liadain Reid, Dag Henrik Reikvam, Renato Reis, Jordi Rello, Jonathan Remppis, Martine Remy, Hongru Ren, Hanna Renk, Anne-Sophie Resseguier, Matthieu Revest, Oleksa Rewa, Luis Felipe Reyes, Tiago Reyes, Maria Ines Ribeiro, Antonia Ricchiuto, David Richardson, Denise Richardson, Laurent Richier, Siti Nurul Atikah Ahmad Ridzuan, Jordi Riera, Ana L. Rios, Asgar Rishu, Patrick Rispal, Karine Risso, Maria Angelica Rivera Nuñez, Nicholas Rizer, Chiara Robba, André Roberto, Stephanie Roberts, David L. Robertson, Olivier Robineau, Ferran Roche-Campo, Paola Rodari, Simão Rodeia, Julia Rodriguez Abreu, Bernhard Roessler, Claire Roger, Pierre-Marie Roger, Emmanuel Roilides, Amanda Rojek, Juliette Romaru, Roberto Roncon-Albuquerque, Mélanie Roriz, Manuel Rosa-Calatrava, Michael Rose, Dorothea Rosenberger, Nurul Hidayah Mohammad Roslan, Andrea Rossanese, Matteo Rossetti, Sandra Rossi, Bénédicte Rossignol, Patrick Rossignol, Stella Rousset, Carine Roy, Benoît Roze, Desy Rusmawatiningtyas, Clark D. Russell, Maeve Ryan, Maria Ryan, Mazankowski Heart Institute Ryckaert, Steffi Ryckaert, Aleksander Rygh Holten, Isabela Saba, Luca Sacchelli, Sairah Sadaf, Musharaf Sadat, Valla Sahraei, Nadia Saidani, Maximilien Saint-Gilles, Pranya Sakiyalak, Nawal Salahuddin, Leonardo Salazar, Jodat Saleem, Nazal Saleh, Gabriele Sales, Stéphane Sallaberry, Charlotte Salmon Gandonniere, Hélène Salvator, Olivier Sanchez, Xavier Sánchez Choez, Kizy Sanchez de Oliveira, Angel Sanchez-Miralles, Vanessa Sancho-Shimizu, Gyan Sandhu, Zulfiqar Sandhu, Pierre-François Sandrine, Oana Sandulescu, Marlene Santos, Shirley Sarfo-Mensah, Bruno Sarmento Banheiro, Iam Claire E. Sarmiento, Benjamine Sarton, Ankana Satya, Sree Satyapriya, Rumaisah Satyawati, Egle Saviciute, Parthena Savvidou, Yen Tsen Saw, Justin Schaffer, Tjard Schermer, Arnaud Scherpereel, Marion Schneider, Stephan Schroll, Michael Schwameis, Gary Schwartz, Janet T. Scott, James Scott-Brown, Nicholas Sedillot, Tamara Seitz, Jaganathan Selvanayagam, Mageswari Selvarajoo, Caroline Semaille, Malcolm G. Semple, Rasidah Bt Senian, Eric Senneville, Claudia Sepulveda, Filipa Sequeira, Tânia Sequeira, Ary Serpa Neto, Pablo Serrano Balazote, Ellen Shadowitz, Syamin Asyraf Shahidan, Mohammad Shamsah, Anuraj Shankar, Shaikh Sharjeel, Pratima Sharma, Catherine A. Shaw, Victoria Shaw, Ashraf Sheharyar, Dr. Rajesh Mohan Shetty, Rohan Shetty, Haixia Shi, Nisreen Shiban, Mohiuddin Shiekh, Takuya Shiga, Nobuaki Shime, Hiroaki Shimizu, Keiki Shimizu, Naoki Shimizu, Sally Shrapnel, Pramesh Sundar Shrestha, Shubha Kalyan Shrestha, Hoi Ping Shum, Nassima Si Mohammed, Ng Yong Siang, Jeanne Sibiude, Bountoy Sibounheuang, Atif Siddiqui, Louise Sigfrid, Piret Sillaots, Catarina Silva, Maria Joao Silva, Rogério Silva, Benedict Sim Lim Heng, Wai Ching Sin, Dario Sinatti, Budha Charan Singh, Punam Singh, Pompini Agustina Sitompul, Karisha Sivam, Vegard Skogen, Sue Smith, Benjamin Smood, Coilin Smyth, Michelle Smyth, Morgane Snacken, Dominic So, Tze Vee Soh, Lene Bergendal Solberg, Joshua Solomon, Tom Solomon, Emily Somers, Agnès Sommet, Myung Jin Song, Rima Song, Tae Song, Jack Song Chia, Michael Sonntagbauer, Azlan Mat Soom, Arne Søraas, Camilla Lund Søraas, Alberto Sotto, Edouard Soum, Ana Chora Sousa, Marta Sousa, Maria Sousa Uva, Vicente Souza-Dantas, Alexandra Sperry, Elisabetta Spinuzza, B. P. Sanka Ruwan Sri Darshana, Shiranee Sriskandan, Sarah Stabler, Thomas Staudinger, Stephanie-Susanne Stecher, Trude Steinsvik, Ymkje Stienstra, Birgitte Stiksrud, Eva Stolz, Amy Stone, Adrian Streinu-Cercel, Anca Streinu-Cercel, Samantha Strudwick, Ami Stuart, David Stuart, Decy Subekti, Gabriel Suen, Jacky Y. Suen, Asfia Sultana, Charlotte Summers, Dubravka Supic, Deepashankari Suppiah, Magdalena Surovcová, Suwarti Suwarti, Andrey A. Svistunov, Sarah Syahrin, Konstantinos Syrigos, Jaques Sztajnbok, Konstanty Szuldrzynski, Shirin Tabrizi, Fabio S. Taccone, Lysa Tagherset, Shahdattul Mawarni Taib, Ewa Talarek, Sara Taleb, Jelmer Talsma, Renaud Tamisier, Maria Lawrensia Tampubolon, Kim Keat Tan, Le Van Tan, Yan Chyi Tan, Clarice Tanaka, Hiroyuki Tanaka, Taku Tanaka, Hayato Taniguchi, Huda Taqdees, Arshad Taqi, Coralie Tardivon, Pierre Tattevin, M Azhari Taufik, Hassan Tawfik, Richard S. Tedder, Tze Yuan Tee, João Teixeira, Sofia Tejada, Marie-Capucine Tellier, Sze Kye Teoh, Vanessa Teotonio, François Téoulé, Pleun Terpstra, Olivier Terrier, Nicolas Terzi, Hubert Tessier-Grenier, Adrian Tey, Alif Adlan Mohd Thabit, Anand Thakur, Zhang Duan Tham, Suvintheran Thangavelu, Vincent Thibault, Simon-Djamel Thiberville, Benoît Thill, Jananee Thirumanickam, Shaun Thompson, David Thomson, Emma C. Thomson, Surain Raaj Thanga Thurai, Duong Bich Thuy, Ryan S. Thwaites, Andrea Ticinesi, Paul Tierney, Vadim Tieroshyn, Peter S. Timashev, Jean-François Timsit, Bharath Kumar Tirupakuzhi Vijayaraghavan, Noémie Tissot, Jordan Zhien Yang Toh, Maria Toki, Kristian Tonby, Sia Loong Tonnii, Antoni Torres, Margarida Torres, Rosario Maria Torres Santos-Olmo, Hernando Torres-Zevallos, Michael Towers, Tony Trapani, Théo Treoux, Huynh Trung Trieu, Cécile Tromeur, Ioannis Trontzas, Tiffany Trouillon, Jeanne Truong, Christelle Tual, Sarah Tubiana, Helen Tuite, Jean-Marie Turmel, Lance C. W. Turtle, Anders Tveita, Pawel Twardowski, Makoto Uchiyama, P G Ishara Udayanga, Andrew Udy, Roman Ullrich, Alberto Uribe, Asad Usman, Timothy M. Uyeki, Cristinava Vajdovics, Luís Val-Flores, Piero Valentini, Ana Luiza Valle, Amélie Valran, Ilaria Valzano, Stijn Van de Velde, Marcel van den Berge, Machteld Van der Feltz, Job van der Palen, Paul van der Valk, Nicky Van Der Vekens, Peter Van der Voort, Sylvie Van Der Werf, Marlice van Dyk, Laura van Gulik, Jarne Van Hattem, Carolien van Netten, Gitte Van Twillert, Ilonka van Veen, Noémie Vanel, Henk Vanoverschelde, Pooja Varghese, Michael Varrone, Shoban Raj Vasudayan, Charline Vauchy, Shaminee Veeran, Aurélie Veislinger, Sebastian Vencken, Sara Ventura, Annelies Verbon, James Vickers, José Ernesto Vidal, César Vieira, Deepak Vijayan, Joy Ann Villanueva, Judit Villar, Pierre-Marc Villeneuve, Andrea Villoldo, Nguyen Van Vinh Chau, Gayatri Vishwanathan, Benoit Visseaux, Hannah Visser, Chiara Vitiello, Manivanh Vongsouvath, Harald Vonkeman, Fanny Vuotto, Noor Hidayu Wahab, Suhaila Abdul Wahab, Nadirah Abdul Wahid, Marina Wainstein, Wan Fadzlina Wan Muhd Shukeri, Chih-Hsien Wang, Steve Webb, Jia Wei, Katharina Weil, Tan Pei Wen, Sanne Wesselius, T. Eoin West, Murray Wham, Bryan Whelan, Nicole White, Paul Henri Wicky, Aurélie Wiedemann, Surya Otto Wijaya, Keith Wille, Suzette Willems, Virginie Williams, Evert-Jan Wils, Calvin Wong, Teck Fung Wong, Xin Ci Wong, Yew Sing Wong, Natalie Wright, Gan Ee Xian, Lim Saio Xian, Kuan Pei Xuan, Ioannis Xynogalas, Sophie Yacoub, Siti Rohani Binti Mohd Yakop, Masaki Yamazaki, Yazdan Yazdanpanah, Nicholas Yee Liang Hing, Cécile Yelnik, Chian Hui Yeoh, Stephanie Yerkovich, Touxiong Yiaye, Toshiki Yokoyama, Hodane Yonis, Obada Yousif, Saptadi Yuliarto, Akram Zaaqoq, Marion Zabbe, Kai Zacharowski, Masliza Zahid, Maram Zahran, Nor Zaila Binti Zaidan, Maria Zambon, Miguel Zambrano, Alberto Zanella, Konrad Zawadka, Nurul Zaynah, Hiba Zayyad, Alexander Zoufaly, David Zucman, Esteban Garcia-Gallo, Laura Merson, Kalynn Kennon, Sadie Kelly, Barbara Wanjiru Citarella, Daniel Vidali Fryer, Sally Shrapnel, James Lee, Sara Duque, Yuli V. Fuentes, Valeria Balan, Sue Smith, Jia Wei, Bronner P. Gonçalves, Clark D. Russell, Louise Sigfrid, Andrew Dagens, Piero L. Olliaro, Joaquin Baruch, Christiana Kartsonaki, Jake Dunning, Amanda Rojek, Aasiyah Rashan, Abi Beane, Srinivas Murthy, Luis Felipe Reyes

**Affiliations:** 1 Universidad de La Sabana, Chía, Colombia; 2 International Severe Acute Respiratory and Emerging Infections Consortium (ISARIC), University of Oxford, Oxford, United Kingdom; 3 Infectious Diseases Data Observatory (IDDO), University of Oxford, Oxford, United Kingdom; 4 The University of Queensland, Brisbane, Australia; 5 The Australian Research Council Centre of Excellence for Engineered Quantum Systems, St. Lucia, Australia; 6 the University of Edinburgh Centre for Inflammation Research, Edinburgh, United Kingdom; 7 Nat. Intensive Care Surveillance- M.O.R.U, Colombo, Sri Lanka; 8 Wellcome-CRIT Care Asia- Africa, Nuffield Department of Clinical Medicine, University of Oxford, Oxford, United Kingdom; 9 Division of Critical Care, Department of Pediatrics, Faculty of Medicine, University of British Columbia, Vancouver, Canada; 10 Ziauddin Medical University Clifton Campus, Karachi, Pakistan; 11 King Abdulaziz Medical City, Riyadh, Saudi Arabia; 12 Tuanku Fauziah Hospital, Perlis, Malaysia; 13 Chiba University Hospital, Chiba, Japan; 14 INSERM, Paris, France; 15 CUB-Hopital Erasme, Anderlecht, Belgium; 16 Bar-Ilan University, Ramat Gan, Israel; 17 Tribhuvan University Teaching Hospital, Kathmandu, Nepal; 18 Perelman School of Medicine at the University of Pennsylvania, Philadelphia, USA; 19 Rinku General Medical Center, Osaka, Japan; 20 Uniklinik University Hospital, Frankfurt, Germany; 21 University Hospital of Parma, Parma, Italy; 22 Centro Hospitalar Vila Nova de Gaia/Espinho, Espinho, Portugal; 23 King Faisal Hospital Research Center, Riyadh, Saudi Arabia; 24 University Hospital, Kerry, Ireland; 25 St Vincents University Hospital, Dublin, Ireland; 26 Murni Teguh Memorial Hospital and Bunda Thamrin Hospital, North Sumatera, Indonesia; 27 Our lady of Lourdes Drogheda, Drogheda, Ireland; 28 Hamad General Hospital, Doha, Qatar; 29 Centre Hospitalier de Saintonge, Saintes, France; 30 Teine Keijinkai Hospital, Sapporo, Japan; 31 Persahabatan Hospital, Jakarta, Indonesia; 32 Al-Adan Hospital, Hadiya, Kuwait; 33 Al-Amiri & Jaber Al-Ahmed Hospitals, Kuwait City, Kuwait; 34 Beaumont Hospital, Dublin, Ireland; 35 Northwell Health, New York, USA; 36 Galway University Hospital, Galway, Ireland; 37 Hospital Vall d’Hebron, Barcelona, Spain; 38 University Hospital Policlinico Paolo Giaccone, Palermo, Italy; 39 ISARIC4C, England, United Kingdom; 40 Centre Hospitalier Universitaire Rouen (Center Hospitalier Universitaire de Rouen), Rouen, France; 41 St Bernard’s Hospital, Gibraltar, Gibraltar; 42 Sligo University Hospital (Saolta), Sligo, Ireland; 43 Hameed Latif Hospital, Lahore, Pakistan; 44 Centre Hospitalier Universitaire de Lille, Lille, France; 45 Centre Hospitalier Universitaire de Nantes (Hôpital femme-enfant-adolescent), Nantes, France; 46 Centre Hospitalier Félix-Guyon, Saint-Denis, Réunion; 47 Abbasi Shaheed Hospital, Karachi, Pakistan; 48 Hospital Universiti Sains Malaysia, Kota Bharu, Malaysia; 49 Centro Hospital e Universitário de Coimbra, Coimbra, Portugal; 50 Hospital de São José -U.U.M., Lisbon, Portugal; 51 Hospital Curry Cabral - Intensive Care Unit -, UCIP7 Lisbon, Portugal; 52 Sungai Buloh Hospital, Selangor, Malaysia; 53 Catharina Ziekenhuis, Eindhoven, Netherlands; 54 The Ottawa Hospital, Ottawa, Canada; 55 Monaldi Hospital, Napoli, Italy; 56 Ospedale Sacro Cuore Don Calabria, Negrar Di Valpolicella, Italy; 57 Centre Hospitalier Universitaire de Brest, Brest, France; 58 Kuala Lumpur Hospital, WPKL, Kuala Lumpur, Malaysia; 59 Fondazione Policlinico Universitario Agostino Gemelli IRCCS, Rome, Italy; 60 Centro de Pesquisa Aggeu Magalhães, Fiocruz, Recife, Brazil; 61 NICVD Dhaka, Dhaka, Bangladesh; 62 National Cardiovascular Center Harapan Kita Jakarta Indonesia, Jakarta, Indonesia; 63 Centro Hospitalar Universitário do Porto (CHUP), Porto, Portugal; 64 Instituto Nacional Del Tórax, Santiago, Chile; 65 Istituto Mediterraneo per i Trapianti e Terapie ad Alta Specializzazione, Palermo, Italy; 66 CISSS Chaudière-Appalaches, Sainte-Marie, Canada; 67 LMU Hospital Munich, Medical Department II, Campus Großhadern, Munich, Germany; 68 Hôpital Européen Georges-Pompidou AP-HP, Paris, France; 69 Groote Schuur Hospital, Cape Town, South Africa; 70 Hospital Ramon y Cajal, Madrid, Spain; 71 University of Iowa, Iowa City, USA; 72 St. Boniface Hospital, Manitoba, Canada; 73 Critical Care Asia Network, Bangkok, Thailand; 74 Nepal Mediciti Hospital, Lalitpur, Nepal; 75 Fujieda Municipal General Hospital, Fujieda, Japan; 76 Hospital Puerta de Hierro Majadahonda, Madrid, Spain; 77 Lao-Oxford-Mahosot Hospital-Wellcome Trust Research Unit, Vientiane, Laos; 78 Luang Namtha Provincial Hospital, Luang Namtha, Laos; 79 Salavan Provincial Hospital, Salavan, Laos; 80 Xieng Khouang Provincial Hospital, Phonsavan, Laos; 81 South City Hospital Karachi, Karachi, Pakistan; 82 North West General Hospital, Peshawar, Pakistan; 83 Centre Hospitalier intercommunal de Créteil, Créteil, France; 84 McGill University Health Centre, Montreal, Canada; 85 Centre Hospitalier de Cholet, Cholet, France; 86 Centre Hospitalier de Perpignan, Perpignan, France; 87 Centre Hospitalier de Dax - Côte d’Argent, Dax, France; 88 The Norwegian Corona Cohort, Oslo, Norway; 89 Hôpital Lariboisière AP-HP, Paris, France; 90 Hôpital Cochin AP-HP, Paris, France; 91 Centre Hospitalier Intercommunal Villeneuve-Saint-Georges, Villeneuve-Saint-Georges, France; 92 Grande Prairie Queen Elizabeth II, Grande Prairie, Canada; 93 WHO-ISARIC Clinical Characterisation Protocol & SPRINT-SARI Collaboration, Oxford, United Kingdom; 94 Pratama Rada Bolo Hospital, Karitas Hospital and Waikabubak Hospital, Sumba, Indonesia; 95 Rush University Medical Center, Chicago, USA; 96 Sotiria General Hospital, Athens, Greece; 97 Unidade Local de Saúde de Alto Minho, Viana Do Castelo, Portugal; 98 ISARIC Global Support Centre, Oxford, United Kingdom; 99 Centre Hospitalier Universitaire de Reims, Reims, France; 100 Instituto de Infectologia Emílio Ribas, Sao Paulo, Brazil; 101 Caja Nacional De Salud, Trinidad, Bolivia; 102 Hospital Universitario de Alava, Araba, Spain; 103 Grand Hôpital de l’Est Francilien (Site de Marne-la-Vallée), Jossigny, France; 104 The Christ Hospital, Ohio, USA; 105 Mayo Hospital Lahore (BICU), Lahore, Pakistan; 106 San Martino Hospital, Genoa, Italy; 107 Clinica Valle de Lilli, Valle del Cauca, Colombia; 108 University of Washington Medical Center - Northwest, Seattle, USA; 109 Raja Permaisuri Bainun Hospital, Perak, Malaysia; 110 Medisch Spectrum Twente, Zutphen, Netherlands; 111 Tartu University Hospital, Tartu, Estonia; 112 Hôpital de l’Enfant-Jésus, Quebec, Canada; 113 Sao Camilo Cura D’ars, Fortaleza, Brazil; 114 Centre Hospitalier Universitaire de Lyon - HCL, Lyon, France; 115 Sarawak General Hospital, Sarawak, Malaysia; 116 University of Cincinnati, Cincinnati, USA; 117 Akershus University Hospital, Nordbyhagen, Norway; 118 Hospital 12 de Octubre, Madrid, Spain; 119 Hôpital Raymond-Poincaré, Garches, France; 120 Oklahoma Heart Institute, Oklahoma, USA; 121 Centre Hospitalier Régional et Universitaire de Nancy - Hôpitaux de Brabois, Nancy, France; 122 Hôpital de la Timone, Marseille, France; 123 Ohio State University, Columbus, USA; 124 Ospedale Papa Giovanni XXIII - Bergamo, Bergamo, Italy; 125 All India Institute of Medical Sciences, Rishikesh, India; 126 Thonon-les-Bains, Thonon-les-Bains, France; 127 Civil Hospital Marie Curie, Charleroi, Belgium; 128 Hôpital Lyon Sud - HCL, Lyon, France; 129 The Centre hospitalier universitaire Sainte-Justine, Montreal, Canada; 130 Rio Hortega University Hospital, Valladolid, Spain; 131 Jena University Hospital, Jena, Germany; 132 Centre Hospitalier Universitaire Mitterrand Dijon-Bourgogne, Dijon, France; 133 National Institute for Communicable Diseases, Johannesburg, South Africa; 134 Ziekenhuisgroep Twente, Hengelo, Netherlands; 135 Hôpital Bichat Claude-Bernard AP-HP, Paris, France; 136 University Hospital Dusseldorf, Dusseldorf, Germany; 137 Centre Hospitalier Universitaire de Saint-Étienne, Saint-Étienne, France; 138 Hôpital Avicenne, Bobigny, France; 139 Centre hospitalier de l’université de Montréal, Montreal, Canada; 140 Centre Hospitalier de Bourg-en-Bresse, Bourg-en-Bresse, France; 141 Centre Hospitalier Universitaire de Besançon, Besançon, France; 142 Centre Hospitalier Universitaire Grenoble-Alpes, Grenoble, France; 143 Centre Hospitalier Universitaire de Nantes (Hôtel-Dieu), Nantes, France; 144 Hôpital d’Instruction des Armées Bégin, Saint-Mandé, France; 145 Franciscus Gasthuis & Vlietland, Rotterdam, Netherlands; 146 National Institute of Infectious Disease Evandro Chagas, Oswaldo Cruz Foundation (INI-FIOCRUZ), Ministry of Health, and D’Or Institute of Research and Education (IDOR), Rio de Janeiro, Brazil; 147 Centre Hospitalier de Mayotte, Mamoudzou, Mayotte; 148 Hospital Egas Moniz, Lisboa, Portugal; 149 Ospedale Molinette, Torino, Italy; 150 Cork University Hospital, Cork, Ireland; 151 Beacon Hospital, Dublin, Ireland; 152 Nelson Hospital, Nelson, New Zealand; 153 Cleveland Clinic, Weston, USA; 154 Medical University of Vienna, Vienna, Austria; 155 Universidad del Cauca, Cauca, Colombia; 156 Sechenov University, Moscow, Russia; 157 Università Cattolica del Sacro Cuore, Rome, Italy; 158 Monash University, Melbourne, Australia; 159 Clinica Universidad de La Sabana, Chia, Colombia; 160 Centre Hospitalier Universitaire de Martinique, Fort-de-France, Saint Martin France; 161 Centre Hospitalier Régional Metz-Thionville, Metz, France; 162 Emory University Healthcare System, Atlanta, USA; 163 Johns Hopkins, Baltimore, USA; 164 Comissão de Ética - Unidade Local de Saúde de Matosinhos, Porto, Portugal; 165 Presbyterian Hospital Services, Alberquerque, USA; 166 Hospital del Mar, Barcelona, Spain; 167 Reina Sofia University Hospital, Cordoba, Spain; 168 Hospital Espírito Santo de Évora, Évora, Portugal; 169 Hôpital Américain de Paris, Neuilly-sur-Seine, France; 170 Vancouver Island Health, Vancouver, Canada; 171 Centre Hospitalier Métropole Savoie, Chambéry, France; 172 University Hospital - Limerick, Limerick, Ireland; 173 Centro Hospitalar e Universitário de Coimbra - Hospital Pediátrico, Coimbra, Portugal; 174 Centre Hospitalier de Béziers, Béziers, France; 175 Hospital São Francisco Xavier, Lisbon, Portugal; 176 Policlinicodi Orsola Universitàdi Bologna, Bologna, Italy; 177 Hospital du Sacre Coeur, Montreal, Canada; 178 Hospital Universitari Sagrat Cor, Barcelona, Spain; 179 Centre Hospitalier de Melun, Melun, France; 180 Sunnybrook Health Sciences Centre, Toronto, Canada; 181 Hôpital Kremlin-Bicêtre, Le Kremlin-Bicêtre, France; 182 Centre Hospitalier Universitaire Rennes (Hôpital Pontchaillou), Rennes, France; 183 Hôpital Tenon AP-HP, Paris, France; 184 Pakistan Kidney & Liver Institute, Lahore, Pakistan; 185 University of Guadalajara Health Sciences Center, Guadalajara, Mexico; 186 National Taiwan University Hospital, Taipei City, Taiwan; 187 Hôpital Saint-Antoine AP-HP, Paris, France; 188 National Institutes of Health (NIH), Ministry of Health Malaysia, Setia Alam, Malaysia; 189 Ospedale San Paolo, Milan, Italy; 190 Chonnam National University Hospital, Dong-gu, South Korea; 191 Pulau Pinang Hospital, Pulau Pinang, Malaysia; 192 Sunway Medical Centre, Selangor, Malaysia; 193 University Hospital Virgen del Rocío/Institute of Biomedicine of Seville, Seville, Spain; 194 University of Utah, Salt Lake City, USA; 195 Children’s Health Ireland, Dublin, Ireland; 196 Foothills Medical Centre, Calgary, Canada; 197 Connolly Hospital Blanchardstown, Dublin, Ireland; 198 Carilion Clinic, Roanoke, USA; 199 Centre Hospitalier Départemental Vendée, La Roche-sur-Yon, France; 200 Allegheny General Hospital, Pittsburgh, USA; 201 Fondazione IRCCS Ca, Milan, Italy; 202 University of Chicago, Chicago, USA; 203 Brantford General Hospital, Brantford, Canada; 204 University of Queensland, Brisbane, Australia; 205 Centre Hospitalier Bretagne Atlantique, Vannes, France; 206 Hôpital Jacques Monod, Le Havre, France; 207 Tergooi Hospital, Hilversum, Netherlands; 208 Michael Garron Hospital, Toronto, Canada; 209 Hospital de Curry Cabral - Infectious Diseases, Lisbon, Portugal; 210 Mount Sinai Medical Center, Miami, FL USA; 211 Azienda Provinciale per i Servizi Sanitari della Provincia Autonoma di Trento, Arco, Italy; 212 Columbia University, New York, USA; 213 Centre Hospitalier Universitaire de Guadeloupe, Pointe-à-Pitre, Guadeloupe; 214 Ospedale Niguarda, Milan, Italy; 215 Centre hospitalier Universitaire de Sherbrooke, Sherbrooke, Canada; 216 UH Cleveland Hospital, Cleveland, USA; 217 University Hospital - Waterford, Waterford, Ireland; 218 Saint-Martin, Saint-, Martin, Guadeloupe; 219 Leiden University Medical Center, Leiden, Netherlands; 220 Centro Hospitalar de Tondela-Viseu, Viseu, Portugal; 221 St Christopher’s Hospital for Children, Philadelphia, USA; 222 Piedmont Atlanta Hospital, Atlanta, Georgia USA; 223 Hôpital Purpan, Toulouse, France; 224 Hôpital Saint-Louis AP-HP, Paris, France; 225 Centre Hospitalier Emile Roux, Le Puy-en-Velay, France; 226 Hôpital Bel-Air, Thionville, France; 227 Centre Hospitalier Universitaire Toulouse (IUCT), Toulouse, France; 228 Alrijne Hospital, Leiden, Netherlands; 229 Policlinico of Padova, Padova, Italy; 230 Centre Hospitalier Universitaire de Nice (Hôpital Archet), Nice, France; 231 Hôpital Albert Calmette, Lille, France; 232 Universitair Ziekenhuis, Gent, Belgium; 233 INOVA Fairfax Medical Center, Fairfax, Virginia USA; 234 Hospital Universitario Dr Negrín, Las Palmas, Spain; 235 Hospital Professor Doutor Fernando Fonseca, Amadora, Portugal; 236 Clinica Las Condes, Santiago, Chile; 237 University Medical Center Groningen, Groningen, Netherlands; 238 Centre Hospitalier Mont-de-Marsan, Mont-de-Marsan, France; 239 Rambam Hospital, Haifa, Israel; 240 Centre Hospitalier Andrée Rosemon, Cayenne, French Guiana; 241 Tallaght University Hospital, Dublin, Ireland; 242 Lions Gate Hospital, Vancouver, Canada; 243 Flevoziekenhuis, Almere, Netherlands; 244 St James’s Hospital, Dublin, Ireland; 245 St Joseph’s Health Center, Sherbrooke, Canada; 246 Centre Hospitalier Universitaire d’Angers, Angers, France; 247 Houston Methodist Hospital, Houston, Texas USA; 248 Rochester General Hospital, New York, USA; 249 Oslo University Hospital, Oslo, Norway; 250 Cleveland Clinic, Ohio, Ohio, OH USA; 251 Medical College of Wisconsin, Wisconsin, USA; 252 Hôpital de la Conception, Marseille, France; 253 Centre Hospitalier de Tourcoing, Tourcoing, France; 254 Reinier de Graaf Gasthuis, Delft, Netherlands; 255 Centre Hospitalier Universitaire Rennes (Hôpital Sud), Rennes, France; 256 Tohoku Medical and Pharmaceutical University, Sendai, Japan; 257 Mar del Plata Medical Foundation Private Community Hospital, Mar Del Plata, Argentina; 258 Long COVID India - Terna Specialty Hospital and Research Centre, Mumbai, India; 259 Hospitales Puerta de Hierro, Jalisco, Mexico; 260 Canisius Wilhelmina Ziekenhuis, Nijmenjen, Netherlands; 261 Hôpital Pellegrin, Bordeaux, France; 262 Centre Hospitalier Pierre Oudot, Bourgoin-Jallieu, France; 263 North York General Hospital, Toronto, Canada; 264 Doctors Hospital, Lahore, Pakistan; 265 Adult ICU Saiful Anwar Hospital, Malang, Indonesia; 266 University of California San Francisco - Fresno, Fresno, USA; 267 Hospital Santa Maria, Centro Hospitalar Universitário Lisboa Norte, Amadora, Portugal; 268 Centre Hospitalier Techer, Calais, France; 269 Centre Hospitalier Régional et Universitaire de Tours, Tours, France; 270 University of Kansas Medical Center, Kansas, USA; 271 The Montreal Children’s Hospital, Montreal, Canada; 272 Vancouver General Hospital, Vancouver, Canada; 273 Ospedale San Gerardo, Monza, Italy; 274 Hôpital Foch, Suresnes, France; 275 Bon Secours Hospital, Cork, Ireland; 276 Hospital Verge de la Cinta, Tortosa, Spain; 277 Hospital Escola da Universidade Federal de Pelotas, Pelotas, Brazil; 278 Saiseikai Senri Hospital, Tochigi, Japan; 279 Manipal Hospital Whitefield, Bangalore, India; 280 RSUP Fatmawati, South Jakarta, Indonesia; 281 Centre Hospitalier de Pau, Pau, France; 282 Hôpital privé d’Antony, Antony, France; 283 Institut Universitaire de Cardiologie et de Pneumologie de Québec, Quebec City, Canada; 284 São João Hospital Centre, Porto, Portugal; 285 San Pedro de Alcantara Hospital, Cáceres, Spain; 286 Beth Israel Deaconess Medical Center, Boston, USA; 287 Ochsner Clinic Foundation, New Orleans, USA; 288 Chitwan Medical College, Chitwan, Nepal; 289 Lugansk State Medical University - Department of Internal Medicine No2, Lugansk, Ukraine; 290 Klinikum Passau, Germant, Germany; 291 Avera McKennan Hospital & University Health Center, Sioux Falls, South Dakota USA; 292 Cleveland Clinic Abu Dhabi, Abu Dhabi, United Arab Emirates; 293 University of Padua, Padua, Italy; 294 Children’s Hospital of Eastern Ontario, Ottawa, Canada; 295 Mater Misericordiae University, Dublin, Ireland; 296 Centre Hospitalier Henri Duffaut, Avignon, France; 297 Mount Kenya University, Thika, Kenya; 298 Hasan Sadikin Hospital, Bandung, Indonesia; 299 The Baruch Padeh Medical Center Poriya, Tiberias, Israel; 300 Lankenau Institute of Medical Research, Wynnewood, USA; 301 The Hospital for Sick Children (SickKids), Toronto, Canada; 302 University Hospital of Tubingen, Tubingen, Germany; 303 Permai Hospital, Johor, Malaysia; 304 University of Michigan Schools of Medicine & Public Health, AnnArbor, USA; 305 Hospital Garcia de Orta, Almada, Portugal; 306 Wexford General Hospital, Wexford, Ireland; 307 Baylor Scott & White Health, Temple, USA; 308 Clinica Alemana DeSantiago, Santiago, Chile; 309 Centre Hospitalier du Pays d’Aix, Aix-en-Provence, France; 310 Centre Hospitalier Universitaire Ambroise-Paré, Boulogne-Billancourt, France; 311 Grigore T Popa University of Medicine and Pharmacy, Bucharest, Romania; 312 Erasmus Medical Centre, Rotterdam, Netherlands; 313 University Children’s Hospital, University Medical Center Hamburg-Eppendorf, Hamburg, Germany; 314 Hospital de Curry Cabral - Internal Medicine, Lisbon, Portugal; 315 Azienda Ospedaliero Universitario Pisana, Pisa, Italy; 316 Centre Hospitalier Universitaire Toulouse (Larrey), Toulouse, France; 317 Hospital de Amor, Sao Paulo, Brazil; 318 Middlemore Hospital (Canties Manukan Health), Otahuhu, New Zealand; 319 Centre Hospitalier de Soissons, Soissons, France; 320 UT Southwestern, Dallas, USA; 321 SIUT Hospital, Karachi, Pakistan; 322 Red Deer Regional Hospital, Red Deer, Canada; 323 Lady Reading hospital, Peshawar, Pakistan; 324 McLeod Healthcare System, Florence, USA; 325 Providence Saint John’s Health Centre, Santa Monica, USA; 326 Kluang Hospital, Johor, Malaysia; 327 Kintampo Health Research Centre, Kintampo, Ghana; 328 University Malaya Medical Centre, Kuala Lumpur, Malaysia; 329 AZ Maria Middelares, Gent, Belgium; 330 Drammen Hospital, Drammen, Norway; 331 Universidad de Las Américas, Quito, Ecuador; 332 University of Maryland, Baltimore, USA; 333 Lancaster General Health, Pennsylvania, USA; 334 PICU Saiful Anwar Hospital, Malang, Indonesia; 335 Nagoya University Hospital, Nagoya, Japan; 336 Centre Hospitalier Le Mans, Le Mans, France; 337 Sultanah Bahiyah Hospital, Kedah, Malaysia; 338 Tuanku Ja’afar, Negeri Sembilan, Malaysia; 339 University of Alabama at Birmingham Hospital, Birmingham, USA; 340 Hokkaido University Hospital, Hokkaido, Japan; 341 Fukuoka University, Fukuoka, Japan; 342 US NHLBI PETAL Network, Boston, USA; 343 University of California - San Francisco (UCSF), San Francisco, USA; 344 Centre Hospitalier Annecy Genevois, Annecy, France; 345 NICVD, Karachi, Pakistan; 346 PIMS, Islamabad, Pakistan; 347 Hippokration Hospital, Thessaloniki, Greece; 348 Hiroshima University, Hiroshima, Japan; 349 Mie University Hospital, Tsu, Japan; 350 Hospital Aleman, Buenos Aires, Argentina; 351 Mills Memorial Hospital, Terrace, Canada; 352 Raja Perempuan Zainab II Hospital, Kelantan, Malaysia; 353 Catholic University, Quito, Ecuador; 354 Hospital Nuestra Señora de Gracia, Zaragoza, Spain; 355 Centre Hospitalier Universitaire Amiens-Picardie, Amiens, France; 356 Sentara Norfolk General Hospital, Norfolk, USA; 357 Kyung Pook National University Chilgok Hospital, Daegu, South Korea; 358 Consortium IMGEN, Piaseczno, Poland; 359 Tawau Hospital, Sabah, Malaysia; 360 Melaka Hospital, Melaka, Malaysia; 361 ABC Hospital, Visakhapatnam, India; 362 Princess Margaret Hospital, Kwai Hung, China; 363 Sanglah General Hospital (Paediatric), Bali, Indonesia; 364 Nationwide Children’s Hospital, Columbus, USA; 365 Shizuoka Children’s Hospital, Shizuoka, Japan; 366 Washington University in StLouis, St Louis, Missouri USA; 367 University of Oklahoma Health Sciences Center, Oklahoma, USA; 368 Groupe Hospitalier Diaconesses Croix Saint-Simon, Paris, France; 369 Hospital for Tropical Diseases, Ho Chi Minh City, Vietnam; 370 Unity Health Toronto, Toronto, Canada; 371 Grande International Hospital, Kathmandu, Nepal; 372 St. Joseph’s Healthcare Hamilton, Hamilton, Canada; 373 Lahad Datu Hospital, Sabah, Malaysia; 374 University Hospital of North Norway, Tromso, Norway; 375 Keimyung University Dong San Hospital, Daegu, South Korea; 376 Kimitsu Chuo Hospital, Chiba, Japan; 377 Hospital for Advanced Medicine and Surgery (HAMS) 1, Kathmandu, Nepal; 378 Obihiro-Kosei General Hospital, Obihiro, Japan; 379 Spaarne Gasthuis, Haarlem, Netherlands; 380 Marmara University Hospital, Istanbul, Turkey; 381 Kharkiv Regional Clinical Infectious Diseases Hospital, Kharkiv, Ukraine; 382 University Health Network, Toronto, Canada; 383 Apollo Hospitals Chennai, Chennai, India; 384 Hartford HealthCare, Hartford, USA; 385 University Airlangga Hospital (Paediatric), Surabaya, Indonesia; 386 Royal Alexandra Hospital, Edmonton, Canada; 387 Centre Hospitalier Alpes-Leman, Contamine-sur-Arve, France; 388 Centre Hospitalier Universitaire Toulouse (Rangueil), Toulouse, France; 389 B & B Hospital, Lalitpur, Nepal; 390 Prof Dr R. D. Kandou Central Hospital, Manado, Indonesia; 391 National Hospital & Medical Center, Lahore, Pakistan; 392 Centre Hospitalier Universitaire de Nîmes, Nîmes, France; 393 Wellington Regional Hospital, Wellington, New Zealand; 394 University of Alberta Adult ICU, Edmonton, Canada; 395 Centre Hospitalier Universitaire de Montpellier, Montpellier, France; 396 Centre Hospitalier Universitaire de Poitiers, Poitiers, France; 397 Queen Elizabeth Hospital, Sabah, Malaysia; 398 Severance Hospital, Seoul, South Korea; 399 Hôpital Henri-Mondor, Créteil, France; 400 University Institute of Cardiology and Respirology, Quebec, Canada; 401 Sultanah Nur Zahirah Hospital, Terengganu, Malaysia; 402 Centre Hospitalier Universitaire Gabriel Montpied, Clermont-Ferrand, France; 403 Institute of TB and Lung Diseases, Warsaw, Poland; 404 Waitemata District Health Board, Auckland, New Zealand; 405 Jinnah Hospital, Lahore, Pakistan; 406 Angeles University Foundation Medical Center, Angeles, Philippines; 407 Malawi-Liverpool Wellcome Trust, Lilongwe, Malawi; 408 Saiseikai Utsunomiya Hospital, Tochigi, Japan; 409 University of Florida, Gainesville, USA; 410 Hospital de Clínicas, Buenos Aires, Argentina; 411 Hospital Emergencia Ate Vitarte, Lima, Peru; 412 Port Macquarie Base Hospital, Port Macquarie, Australia; 413 Netcare Unitas ECMO Centre, Centurion, South Africa; 414 Hospital Universitario Virgen de Valme, Seville, Spain; 415 Stanford University, Palo Alto, USA; 416 Klinik und Poliklinik für Innere Medizin II, University Hospital Regensburg, Kiel, Germany; 417 William Osler Health Sciences System - Etobicoke General Hospital, Toronto, Canada; 418 Hôpital Louis-Mourier, Colombes, France; 419 Mercy Hospital, Cork, Ireland; 420 Hospital Vila Franca de Xira, Lisbon, Portugal; 421 La Paz Hospital, Madrid, Spain; 422 Alberta Children’s Hospital, Calgary, Canada; 423 Centre Hospitalier de Colmar, Colmar, France; 424 Kingston Health Sciences Centre, Kingston, Canada; 425 Brooke Army Medical Centre, San Antonio, USA; 426 International Islamic University Malaysia Medical Centre (IIUMMC), Pahang, Malaysia; 427 Hospital Sirio-Libanes, Sao Paulo, Brazil; 428 Waikato Hospital, Hamilton, New Zealand; 429 Auckland City Hospital, Auckland, New Zealand; 430 Mount Sinai Hospital, Toronto, Canada; 431 London Health Sciences Centre, London, Canada; 432 GMMM Teaching Hospital, Sukkar, Pakistan; 433 Lahore General Hospital, Lahore, Pakistan; 434 Centre Hospitalier de Cahors, Cahors, France; 435 Borgo San Lorenzo Hospital, Trento, Italy; 436 Centre Hospitalier Universitaire Rouen (Hôpital Charles Nicolle), Rouen, France; 437 Hospital de Especialidades Eugenio Espejo, Quito, Ecuador; 438 Hospital Clinic, Barcelona, Spain; 439 Tengku Ampuan Afzan Hospital, Pahang, Malaysia; 440 Jinnah Post-Graduate Medical Center (SICU), Karachi, Pakistan; 441 Sinai Health Systems, Toronto, Canada; 442 BC Children’s Hospital, Vancouver, Canada; 443 Darul Sehat Hospital, Karachi, Pakistan; 444 University Hospital Northern British Columbia, Prince George, Canada; 445 St-Pierre University Hospital, Brussels, Belgium; 446 Hospital Da Luz, Lisbon, Portugal; 447 Queen Mary Hospital, Pok Fu Lam, China; 448 Queen Elizabeth Hospital, Yau Ma Tei, China; 449 Siriraj Piyamaharajkarun Hospital (SiPH), Bangkok, Thailand; 450 Oregon Health & Science University, Portland, USA; 451 Department of Children’s Infectious Diseases, Warsaw, Poland; 452 Dr Sardjito Government Hospital, Yogyakarta, Indonesia; 453 Alanda Health, Oslo, Norway; 454 Clínica Pasteur, Neuquén, Argentina; 455 RSUD Pasar Minggu, South Jakarta, Indonesia; 456 Misericordia Community Hospital, Edmonton, Canada; 457 Legacy Emanuel Medical Center, Portland, USA; 458 Instituto do Coração da Universidade de São Paulo (INCOR), São Paulo, Brazil; 459 Joseph Brant Hospital, Burlington, Canada; 460 Tufts Medical Centre, Boston, USA; 461 Mayo Clinic School of Medicine, Arizona, USA; 462 Hospital General San Francisco, Quito, Ecuador; 463 McMaster University, Hamilton, Canada; 464 Azeema Sheikh Hospital, Islamabad, Pakistan; 465 Hospital Beatriz Ângelo, Loures, Portugal; 466 Niagara Health, Niagara, Canada; 467 Ispat General Hospital, Rourkela, India; 468 Centre Hospitalier de Périgueux, Périgueux, France; 469 University Hospital Ostrava, Ostrava-Poruba, Czechia; 470 Humber River Hospital, Toronto, Canada; 471 Maastricht University Medical Centre, Maastricht, Netherlands; 472 University of Brescia, Brescia, Italy; 473 North Estonia Medical Centre, Tallin, Estonia; 474 RSUD Dr. Soetomo, Surabaya, Indonesia; 475 Pushpagiri Medical College Hospital, Kerala, India; 476 Baylor University Medical Centre, Dallas, USA; 477 National University Hospital, Singapore, Singapore; 478 Bahria International Hospital, Islamabad, Pakistan; 479 Hospital de Abrantes - ICU, Abrantes, Portugal; 480 Hôpital Européen Marseille, Marseille, France; 481 Centre Hospitalier Agen-Nérac, Agen, France; 482 Patel Hospital, Karachi, Pakistan; 483 University of Manitoba, Manitoba, Canada; 484 The Center for Diagnosis, Santo Domingo, Dominican Republic; 485 CHU Carémeau, Nimes, France; 486 Mazankowski Heart Institute, Edmonton, Canada; 487 Sheikh Zayed Medical College Rahim yar Khan, Rahim yar Khan, Pakistan; 488 Hôpital Laënnec - site de Quimper, Quimper, France; 489 Fundación Cardiovascular de Colombia, Floridablanca, Colombia; 490 Hospital Universitari Sant Joan D’Alacant, Alicante, Spain; 491 National Institute for Infectious Diseases Matei Bals, Bucharest, Romania; 492 Centro Hospitalar Universitário do Algarve, Portimão, Portugal; 493 RSPI Prof Dr Sulianti Saroso, Jakarta, Indonesia; 494 The Heart Hospital Baylor Plano, Plano, USA; 495 Gelre Hospitals, Zutphen, Netherlands; 496 Krankenhaus Barmherzige Br, Regensburg, Germany; 497 Baylor AllSaints Medical Centre, Fort Worth, USA; 498 Sozialmedizinisches Zentrum Sud, Vienna, Austria; 499 Mehta Hospital, Chennai, India; 500 Centro Hospitalar de Leiria, Leiria, Portugal; 501 Tohoku University, Sendai, Japan; 502 Hyogo Prefectural Kakogawa Medical Center, Hyogo, Japan; 503 Tokyo Metropolitan Tama Medical Center, Tokyo, Japan; 504 St. Marianna University School of Medicine, Kawasaki, Japan; 505 Om Hospital, Kathmandu, Nepal; 506 Karuna Hospital, Kathmandu, Nepal; 507 Pamela Youde Nethersole Eastern Hospital, Chai Wan, China; 508 Grand River Hospital, Kitchener, Canada; 509 Seoul National University Bundang Hospital, Seoul, South Korea; 510 Hospital Naval Marcílio Dias, Rio De Janeiro, Brazil; 511 Hospital Universitário Clementino Fraga Filho, Rio de Janeiro, Brazil; 512 Baerum Sykehus, Gjettum, Norway; 513 Sturgeon Community Hospital, St Albert, Canada; 514 University Hospital in Krakow, Krakow, Poland; 515 Centre Hospitalier Universitaire Grenoble-Alpes_FU, Grenoble, France; 516 Hospital das Clinicas da Faculdade de Medicina da Universidade de Sao Paulo, Sao Paulo, Brazil; 517 Kyoto Medical Centre, Kyoto, Japan; 518 Yokohama City University Medical Center, Yokohama, Japan; 519 Fatmawati Hospital, Jakarta, Indonesia; 520 Complexo Hospitalar DrClementino Fraga, João Pessoa city, Brazil; 521 Nidan Hospital, Lalitpur, Nepal; 522 Centre Hospitalier Louis Raffalli, Manosque, France; 523 University of Nebraska Medical Center, Omaha, USA; 524 Clínica Internacional, Lima, Peru; 525 Hôpital Robert-Debré AP-HP, Paris, France; 526 Dunedin Public Hospital, Dunedin, New Zealand; 527 Mater Dei Hospital, Belo Horizonte, Brazil; 528 ADRZ, Amsterdam, Netherlands; 529 Adrz, Goes, Netherlands; 530 Meander Medical Centre, Amersfoort, Netherlands; 531 Noordwest-Ziekenhuisgroep, DenHelder, Netherlands; 532 Kerala Institute of Medical Sciences, Trivandrum, India; 533 Grey Nun’s Community Hospital, Edmonton, Canada; 534 Beatrix ziekenhuis, Gorinchem, Netherlands; 535 Royal Columbian Hospital, Vancouver, Canada; 536 Kyoto Prefectural University of Medicine, Kyoto, Japan; 537 Kouritu Tousei Hospital, Seto City, Japan; 538 MedStar Washington Hospital Centre, Washington, USA; 539 Sultanah Aminah Hospital, Johor, Malaysia; 540 University of Western Australia/Fiona Stanley Hospital, Murdoch, Australia

**Keywords:** Epidemiology, Viral infection

## Abstract

The International Severe Acute Respiratory and Emerging Infection Consortium (ISARIC) COVID-19 dataset is one of the largest international databases of prospectively collected clinical data on people hospitalized with COVID-19. This dataset was compiled during the COVID-19 pandemic by a network of hospitals that collect data using the ISARIC-World Health Organization Clinical Characterization Protocol and data tools. The database includes data from more than 705,000 patients, collected in more than 60 countries and 1,500 centres worldwide. Patient data are available from acute hospital admissions with COVID-19 and outpatient follow-ups. The data include signs and symptoms, pre-existing comorbidities, vital signs, chronic and acute treatments, complications, dates of hospitalization and discharge, mortality, viral strains, vaccination status, and other data. Here, we present the dataset characteristics, explain its architecture and how to gain access, and provide tools to facilitate its use.

## Abstract (summary)

**Table:** 

Measurement(s)	CDISC SDTM Vital Sign Test Name Terminology
Technology Type(s)	CDISC Define-XML General Observation Class Terminology
Factor Type(s)	Hospital Mortality • COVID-19
Sample Characteristic - Organism	Homo sapiens
Sample Characteristic - Environment	hospital

## Background & Summary

The International Severe Acute Respiratory and Emerging Infection Consortium (ISARIC) is a global federation of clinical research networks collaborating to prevent illness and death from infectious disease outbreaks through proficient and agile research response^1^. In January 2020, ISARIC launched a research response to the emergence of a novel severe acute respiratory syndrome coronavirus (SARS-COV-2), detected weeks earlier in Wuhan, China^2,3^. The initial focus was on the clinical characterisation of COVID-19, the disease caused by SARS-CoV-2, which mainly affects the respiratory system^2^. The fatality rate of COVID-19 varies substantially across different locations, which may reflect differences in population age, comorbidities, vaccination status, and other factors^4^. In June 2022, there were more than 500 million reported cases and more than 6 million deaths. Despite unprecedented success in the rapid generation of vaccines and effective treatments, COVID-19 continues to cause severe and widespread health consequences^5,6^. Therefore, the continuation of high-quality, globally-representative research is critical – as are the data required to deliver it.

**Fig. 1:**
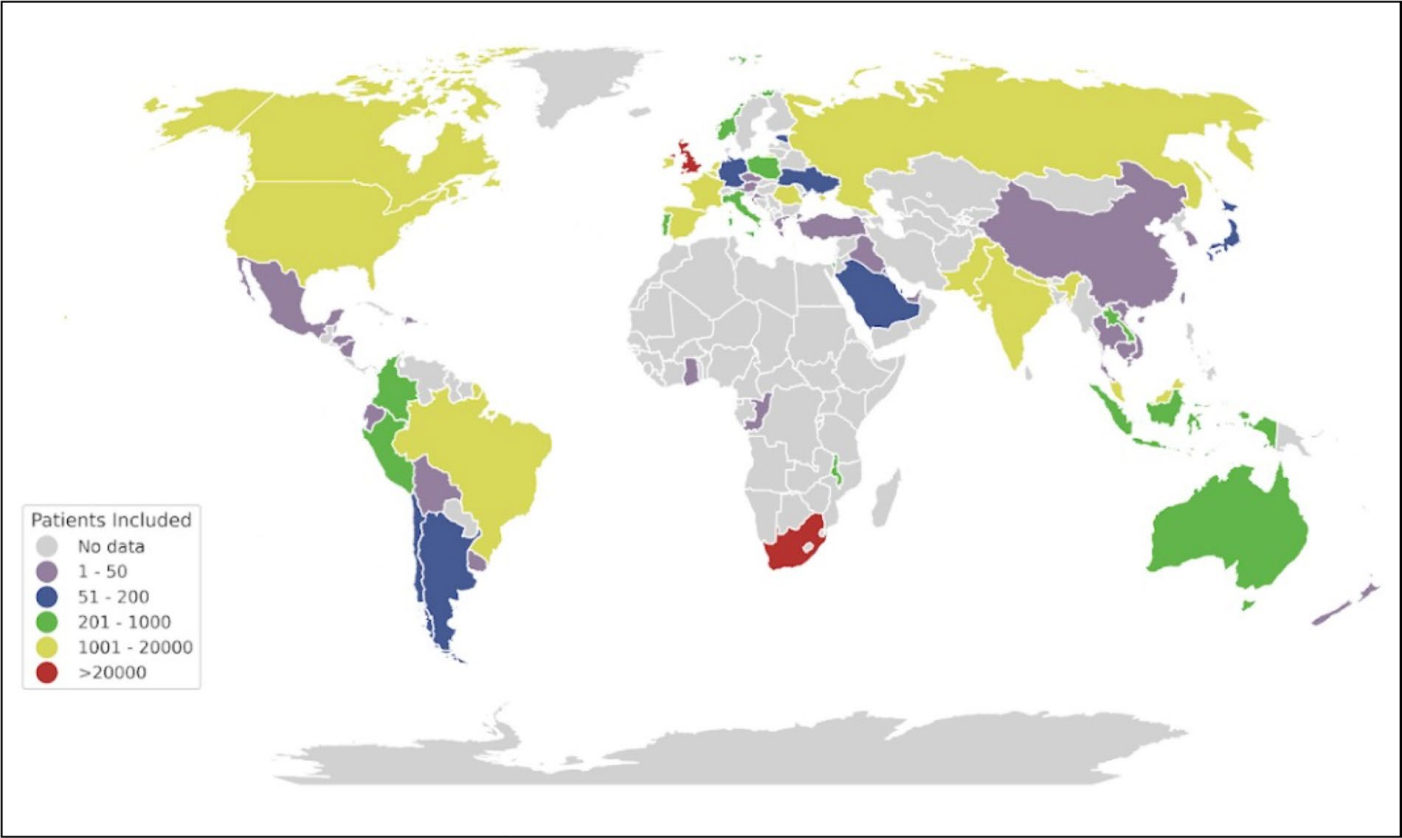
The number of patients per country is included in the ISARIC COVID-19 database.

At the beginning of the COVID-19 outbreak, ISARIC adapted the ISARIC-WHO Clinical Characterization Protocol and data tools^7^ to facilitate global research collaboration and accelerate the understanding of COVID-19 as part of the public health response to the pandemic^1,8,9^. Between January 2020 and September 2021, information about the clinical presentation, treatment, and outcomes of more than 705,000 patients with COVID-19, hospitalized across 62 countries, was aggregated to form the ISARIC-COVID-19 dataset. Clinical teams in 1,559 participating institutions collected the data. Figure 1 shows the number of patients per country included in the database as of September 2021^1,4,10^. The number of patients included in the dataset continues to grow as data collection continues across the globe.

The objective of the dataset is to accelerate understanding of COVID-19 through access to detailed clinical information on infected patients from a range of settings. Access to data facilitates science, improves scientific transparency and integrity, and has played a substantial role in the generation of knowledge that has led to better patient management and vaccine production for COVID-19^11^. The diversity of populations, regions, and resource levels from which the data originate increases the generalizability of the evidence generated and supports comparisons across them. By collating, standardizing, and sharing large volumes of disparate data, curation and governance efforts are invested centrally by a specialised team, enabling efficient data access, and analysis by many researchers focused on the questions most relevant to the patients in their settings. This approach accelerates pandemic response by promoting locally-driven, locally-relevant knowledge generation, which is most likely to have an impact on public health policy and drive societal benefits beyond health^12,13^.

## Methods

### Data collection

Standardized clinical data of patients with suspected or confirmed COVID-19 are collected on the ISARIC-WHO case report forms (CRFs) (https://isaric.org/research/covid-19-clinical-research-resources/covid-19-crf/) or site-specific iterations of these forms. These forms are available in multiple languages to support accessibility for a global response.

Sites implement data collection contemporaneously to clinical care. Data are collected through direct observation and/or reviewing and extracting electronic health records or patient registries. Data can be submitted to ISARIC by completing the CRF on the Research Electronic Data Capture platform (REDCap version 10.6 Vanderbilt University^14^) hosted by the University of Oxford. Alternatively, institutions using other data collection forms and/or a different data management system can share patient data in any format to the ISARIC COVID-19 data platform, hosted by the Infectious Diseases Data Observatory (IDDO, www.iddo.org). Data were prospectively collected on patients with clinical suspicion or laboratory confirmation of SARS-CoV-2 infection and admitted to a participating hospital or ward. Recruitment aimed to include all identified patients; however, resource constraints limited enrolment when patient numbers surged and health systems became overwhelmed. In such cases, or in sites where prospective data collection was impossible, data were extracted from electronic health records. Ethics approval and informed consent were obtained according to local regulations, which included a waiver of consent to collect de-identified data at several sites due to the burden on front-line workers and the data protection framework in place. The WHO-ISARIC Clinical Characterization Protocol was approved by the WHO Ethics Committee (RPC571 and RPC572).

### Data standardization

The ISARIC COVID-19 dataset is a large, clinically comprehensive, international resource. The diversity of data aggregated to create this resource required a uniform data model to standardize the structures and ontologies to a harmonized format. Thus, all data are standardized to the Clinical Data Interchange Standards Consortium (CDISC) Study Data Tabulation Model (SDTM) to facilitate pooled analyses. While there is no perfect data model, the CDISC SDTM was chosen to allow maximum flexibility to accommodate the diverse data types collected by different groups. This was preferred over other options, such as the Observational Medical Outcomes Partnership (OMOP) model, which was more rigid with a fixed number of possible tables and variables. The use of SDTM also allows for greater interoperability to enable integration with COVID-19 clinical trial data that may be added to the dataset in the future. This data model is designed for data tabulation and storage. Using the dataset requires processing to create an analysis dataset from which results can be derived. Here we present a complete description of the available data, how it is formatted, and describe a generalizable strategy to use and maximize its utility in research.

### Data standardization - de-identification

Data entered in the ISARIC REDCap database or uploaded to the IDDO data platform are reviewed to ensure no direct identifiers are included. Direct identifiers, including those listed in the UK General Data Protection Regulation (https://ico.org.uk/for-organisations/guide-to-data-protection/guide-to-the-general-data-protection-regulation-gdpr/) and the US Health Insurance Portability and Accountability Act (https://www.hhs.gov/hipaa/index.html), are permanently deleted before data are curated through various processes.

### Data standardisation - pre-mapping

Data and all documentation shared with the data, such as dictionaries, protocols, publications, and data collection forms, are reviewed by the data curator to fully understand the contents of the dataset. Queries are raised with the data contributor when required. Each variable in the dataset is assigned to the appropriate SDTM domain(s), variable(s), and controlled vocabulary (if applicable) according to the rules found within the IDDO SDTM Implementation Manual (https://www.iddo.org/tools-and-resources/data-tools). The implementation manual chronicles each type of data curated to the platform and is consulted and updated with each new dataset to ensure consistency across the repository. An audit trail of the assignments is also recorded in a dataset-specific SDTM mapping guide.

### Data standardization - data wrangling

For formatting and coding, the contributed datasets are loaded into Trifacta®, a data wrangling programme. This can include merging files, splitting variables into separate domains, applying controlled terminology to variables, and adding created variables as required. IDDO-defined standardization, conversion, and categorization formulas are also used as described in the IDDO SDTM Implementation Manual. Transformations on the contributed data (in the interests of standardization) are recorded and stored in a form that documents the transformation and enables it to be reproduced.

### Data standardization - review and edit checks

Data is run through Pinnacle 21® (community version) software, a CDISC standards compliance-verification tool that checks the standard SDTM implementation guide rules and requirements for regulatory submission. The resulting checks and warnings are assessed for applicability to the individual dataset. The data are also run through standard edit checks to identify possible mapping errors separate from SDTM conformance. The curator adjusts the mapping as needed to make corrections.

**Fig. 2:**
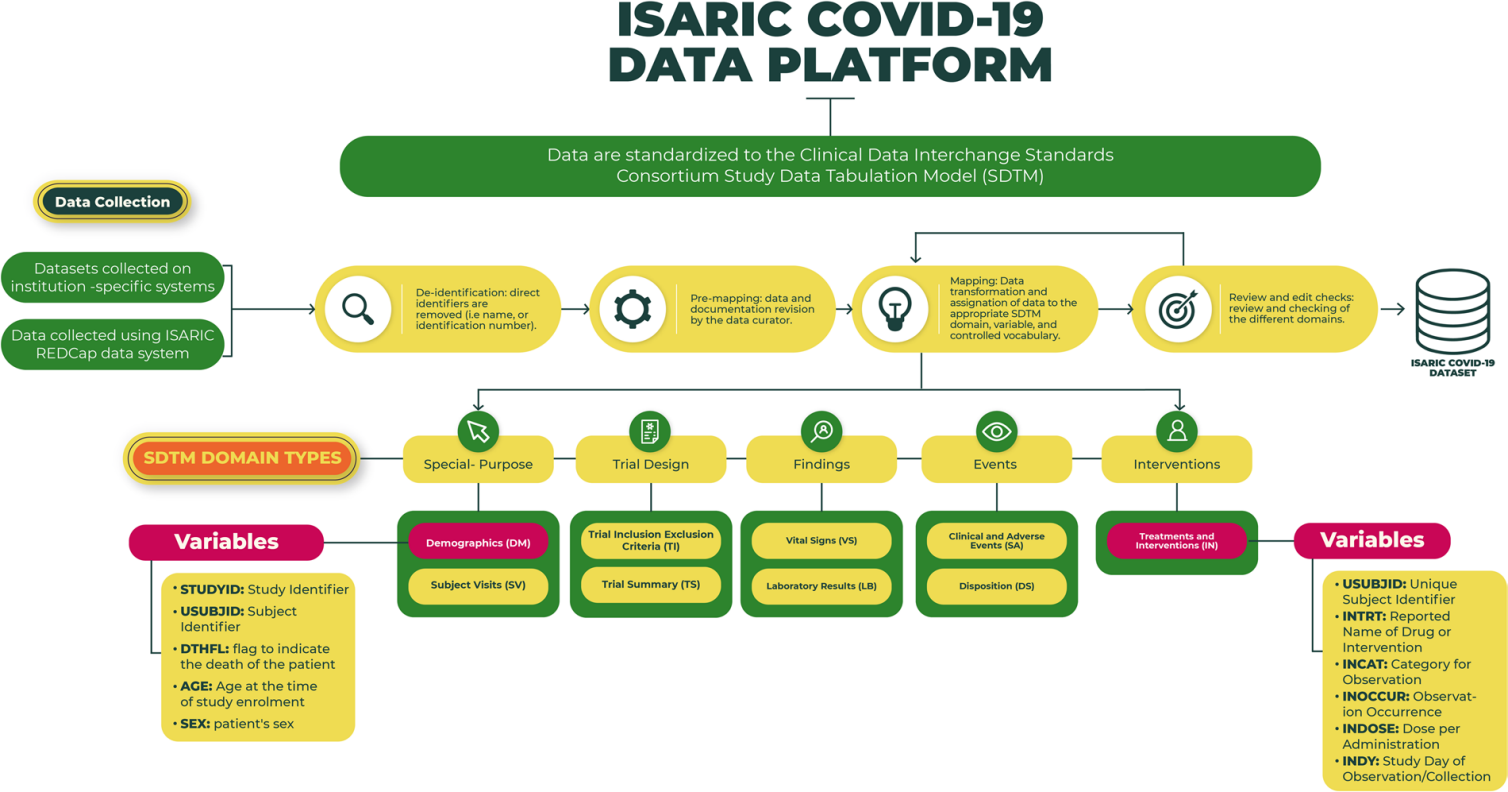
Overview of the ISARIC COVID-19 Database.

Figure 2 describes the workflow from data acquisition to the final, pooled dataset that researchers can access to conduct their research.

## Data Records

The dataset is available from the Infectious Diseases Data Observatory – IDDO at 10.48688/nx85-bv30^15^ The ISARIC-COVID-19 dataset is a relational database consisting of 16 tables, each representing a domain of information set out in the CDISC SDTM data model. Unique identifiers link these with the suffix ‘ID.’ For example, USUBJID refers to the subject’s unique identifier, which is the primary key for assessing individual-level data; STUDYID contains the unique identifier for an individual hospital or network of hospitals. Each table defines and tracks different aspects of illness and treatment.

### Data tables

**Fig. 3:**
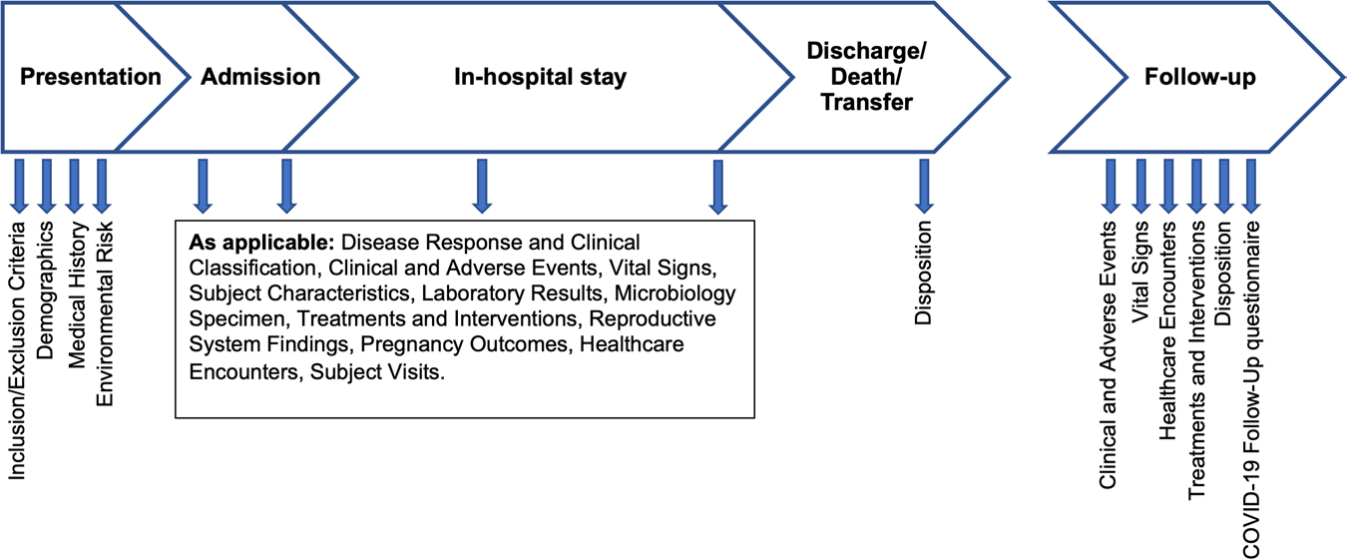
Data collection points for each data type.

**Fig. 4:**
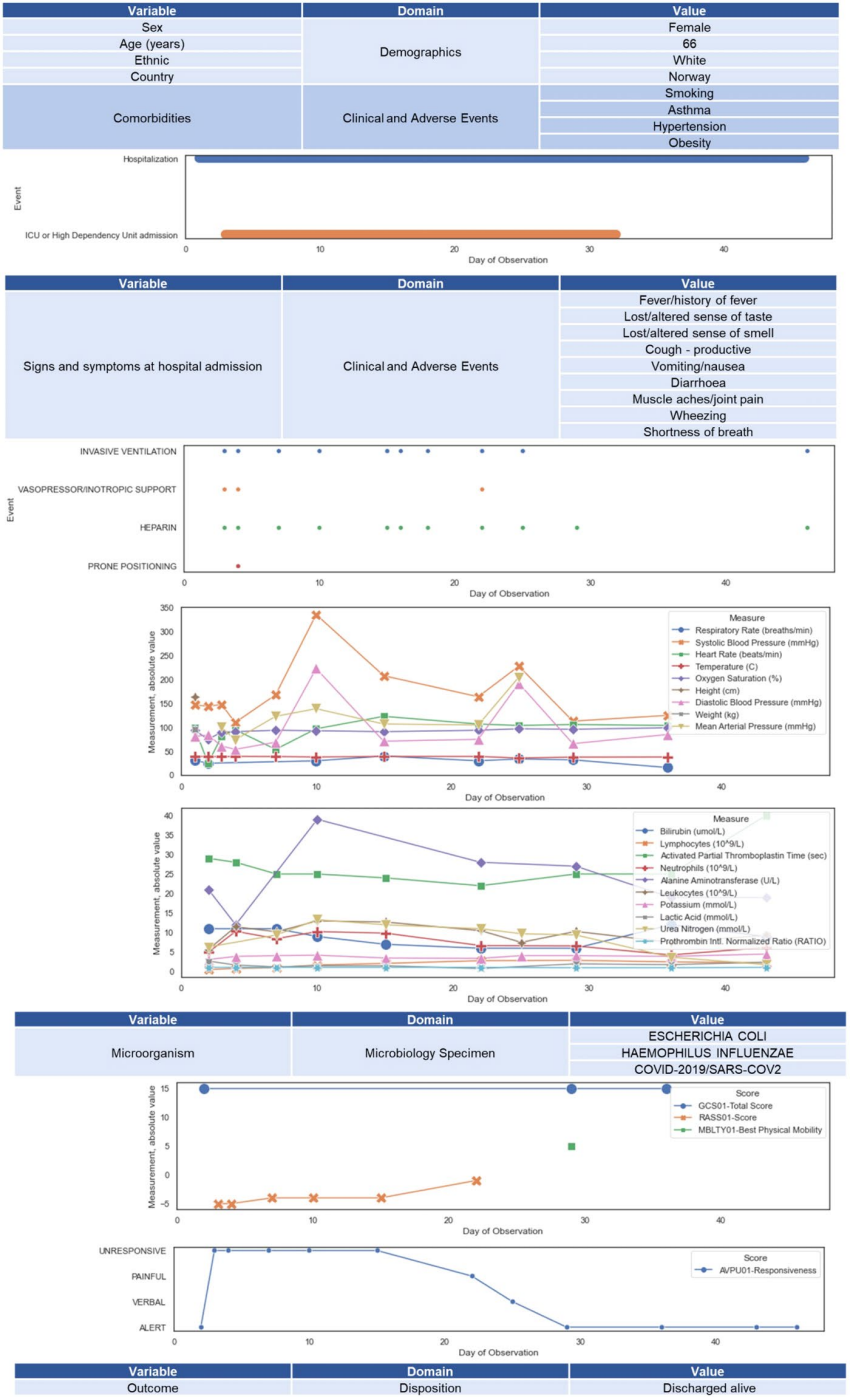
A synthetic, representative subset of the available data for a female patient.

The tables (i.e., domains) currently included in the dataset are Demographics (DM), Disposition (DS), Environmental Risk (ER), Healthcare Encounters (HO), Inclusion/Exclusion Criteria (IE), Treatments and Interventions (IN), Laboratory Results (LB), Microbiology Specimen (MB), Reproductive System Findings (RP), Disease Response and Clinical Classification (RS), Clinical and Adverse Events (SA), Subject Visits (SV), Vital Signs (VS), COVID-19 Follow-Up questionnaire (CQ), Subject Characteristics (SC), and Pregnancy Outcomes (PO) (Supplementary Table 1); The majority of those tables are at a patient level, so it has a subject id (USUBJID) that that relates the information of a single patient distributed in the multiple tables. The Trial Summary (TS), Trial Inclusion Exclusion Criteria (TI), and Device Identifiers (DI) are study-level domains; thus, there is no individual patient-level data in those domains. Instead, there is information about the uniqueness of each institution, for instance, the inclusion/exclusion criteria or the devices used at each hospital. Data collection times for each data type are presented in Fig. 3^16–18^. As an example, we show in Fig. 4 a synthetic, representative subset of the available data for a female patient.

The CDISC SDTM data model has several advantages. For example:

1. It can adapt to any number of events. Frequently recorded events such as vital signs, laboratory tests, and patient status scores are stored as a series of events. The order is recorded in the variables with the suffix ‘DY,’ which describes the day of the observation relative to the patient’s hospital admission date. For example, the variable ‘VSDY’ indicates the day when a particular vital sign was measured. Events occurring within the same day can be further ordered using the variables with the suffix ‘SEQ’, which captures the sequence of events independently of the day on which they occurred.
2. It captures whether or not a variable was collected for a given patient (this is critical to count denominators accurately in an aggregated collection of many different datasets). The model enables this by collecting the existence of a variable separately from the occurrence or completion of that variable. E.g., if the CRF for a dataset includes data on fever, the model shows that this question was prespecified as FEVER_PRESP = Yes; if the patient had a fever, it is captured as FEVER_OCCUR = Yes; if the patient was afebrile, it is registered as FEVER_OCCUR = No. Combining these two variables makes it possible to accurately quantify how many patients were evaluated for fever and how many had a fever. This distinction is found in the ER, HE, IN, and SA tables. A full description of how SDTM is implemented for these data, Frequently Asked Questions, and other data tools are available within the IDDO suite of curation and data resources (https://www.iddo.org/tools-and-resources/data-tools) to assist analysts in understanding these nuances. The remaining tables contain study-level data (e.g., Study Inclusion Exclusion Criteria and Device Identifiers); thus, there are no individual-level data in these domains.

The dataset also contains a rich repository of free-text entries that capture more fine-grained information not included in the CRF solicited entries. Such information can be identified by applying simple search functions or Natural Language Processing (NLP) techniques to the **TERM variable. Supplementary Table 1 describes how data is distributed across the domain data tables and how many unique patients are included in each table.

### Patient characteristics

Among the 708,158 patients whose data were entered as of September 2021, 552,366 (78%) had laboratory confirmation of SARS-CoV-2 infection, and 50,426 (7,1%) were clinically diagnosed (where testing was not available or results were not reported). Of these patients, the median age (interquartile ranges: first quartile (Q1) and third quartile (Q3)) is 58 (IQR: 44–72) years, 48.9% are male, and 50.9% are female (the sex of 0.1% of the patients is unknown). A total of 126,069 (20.9%) patients were admitted to a critical care unit (ICU or HDU), and in-hospital mortality was 23.5%^5^. Table 1 provides a breakdown of the population by continent, and Supplementary Table 1 shows the number of unique patients with data reported per each domain.

**Table 1:** Details of the ISARIC-COVID-19 patient population by continent.

Continent	Global	Africa	Europe	Asia	North America	South and Central America	Oceania
	n = 602792	n = 369467	n = 206992	n = 16019	n = 6687	n = 2709	n = 448
Critical care admission, counts (%)	126069 (20.91)	73095 (19.78)	35454 (17.13)	11544 (72.06)	3619 (54.12)	1872 (69.10)	427 (95.31)
Age, years, median (Q1-Q3)	58 (44–72)	54 (40–66)	70 (54–82)	58 (46–68)	64 (52–76)	54 (42–66)	62(51–70)
Male, counts (%)	294928 (48.93)	165376 (44.76)	113148 (54.66)	10366 (64.71)	3857 (57.68)	1659 (61.24)	269 (60.04)
In-hospital mortality, counts (%)	141646 (23.5)	88737 (24.02)	46424 (22.43)	4310 (26.91)	1672 (25)	440 (16.24)	59 (13.17)

The information presented in the table is based on the raw data, and there is missing data, for instance: 470 patients do not have their country of origin registered; 8143 patients do not have age; 149 do not have their sex registered, and the outcome of 10130 patients is missing.

**Fig. 5:**
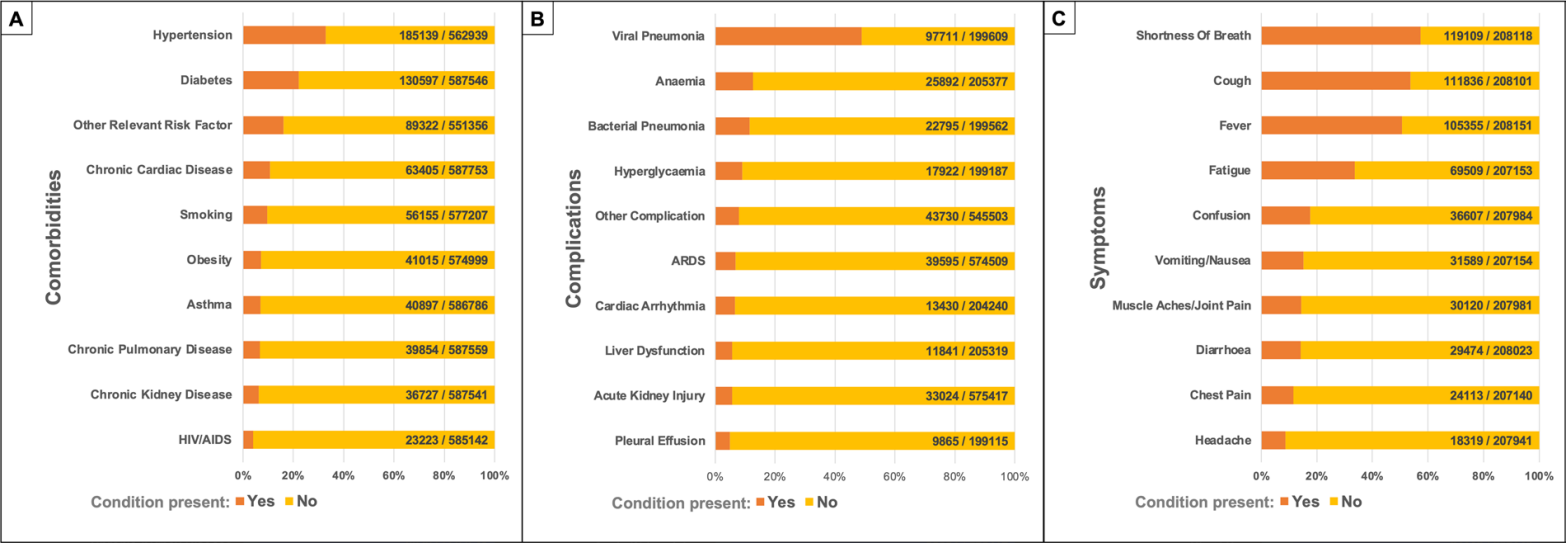
Distribution of primary symptoms, comorbidities, and treatments. (**A**) shows the prevalence of comorbidities; (**B**) shows the prevalence of symptoms at admission; (**C**) shows the proportion of patients receiving each treatment.

The most frequently reported comorbidities, symptoms at hospital admission, and complications during hospital admission are presented in Fig. 5. Among comorbid conditions, hypertension (30.7%), diabetes mellitus (29.6%), and chronic cardiac disease (10.5%) were the most frequently reported. The top five symptoms at admission were cough (23.7%), shortness of breath (19.8%), fever (17.5%), fatigue (11.5%), and altered consciousness (6.1%). Regarding complications, viral pneumonia (16.2%), acute respiratory distress syndrome (6.6%), acute kidney injury (5.5%), anaemia (4.3%), and bacterial pneumonia (3.8%) were the most frequently identified.

## Technical Validation

Data submitted via the ISARIC REDCap system are subjected to a series of field-specific data quality checks designed by ISARIC. These trigger error alerts inform users of issues based on value limits, validate dates, flag missing variables, and perform logic checks to compare related variables. Data are further reviewed by a data manager who sends data quality reports and queries to sites when critical data are missing or outside expected values. Staff at data collection sites review the alerts and make the necessary corrections to their data in the REDCap system.

Data uploaded to the IDDO platform are verified during the ‘pre-mapping’ and ‘data review and edit checks’ processes described above. Interpretation of the data dictionary (for sites that used a unique data collection tool) and any missing values are queried directly with staff at the data collection sites. Results are charted per variable to identify and query outlier values. Where correction is suggested, the contributing site is contacted and asked to correct the data as needed before re-uploading them to the data platform.

## Usage Notes

The utility of the data collected is optimised by issuing regular open-access ISARIC COVID-19 Clinical Data Reports (https://isaric.org/research/covid-19-clinical-research-resources/evidence-reports/) and periodic updates to the ISARIC COVID-19 Dashboard (https://livedataoxford.shinyapps.io/CovidClinicalDataDashboard/). Data are available for analysis through two mechanisms to maximize uptake: a collaborative mechanism for ISARIC partners who contribute data to the dataset and a data-sharing platform for external researchers. The sites that contribute to the data retain ownership and decision-making authority on their data at all times.

It is essential to highlight that more countries are globally transitioning to digital-based healthcare systems. During the transitioning process, quality control measures are necessary to enhance the effectiveness of healthcare-related communication and data quality^19^. Thus, the ISARIC-COVID-19 dataset can generate insights facilitating quality control measures, especially in developing countries where scarce scientific resources.

### Data access

Staff from sites that contribute data to the dataset may access data for collaborative analysis via the ISARIC Partner Analysis scheme (https://isaric.org/research/isaric-partner-analysis-frequently-asked-questions/). Proposals for these analyses are governed and supported by ISARIC and executed with all data contributors’ contributions, oversite, and accreditation^4,10,20^. ISARIC provides statistical, clinical, and administrative support to promote analyses by partners who contribute the data, especially those based in low-resource settings.

External researchers who have not contributed to the dataset are also welcome to submit a data access and analysis proposal via the IDDO platform (https://www.iddo.org/covid19). An independent Data Access Committee reviews these requests according to the Data Access Guidelines of the platform. (https://www.iddo.org/covid19/data-sharing/accessing-data). Statistical analysis plans and outputs from both types of access can be viewed at: https://www.iddo.org/covid19/research/approved-uses-platform-data.

Data management, curation, governance, and the data-sharing platform are free to use and supported by the ISARIC and IDDO data management teams. When shared through the governed data access mechanisms, the ISARIC COVID-19 database is provided as a collection of comma-separated value (CSV) files (i.e., tables), along with scripts to help import the data into PostgreSQL and codes that enable the reuse of the data. Notably, where data transformations are made during the database construction process, care is taken not to modify raw study data. The teams performing analyses can develop analytic codes based on assumptions they deem appropriate.

### Data use

The breadth of analyses published to date demonstrates the diversity of science that can be generated from these data. Examples include identification of unique COVID-19 symptomology at the extremities of age^21^; to develop the ISARIC 4 C mortality score that outperformed existing scores and showed utility to directly inform clinical decision making^22^; to identify temporal trends in inpatient journeys and inform resource needs in an evolving pandemic^10^, and to improve the diagnosis of acute kidney injury^23^. Further analyses to develop natural language processing, understand neurological outcomes in COVID-19 and develop models that predict a range of outcomes.

The use of such a large and diverse dataset is not without challenges. Robust interpretation of analytic outputs requires an understanding of the variation in recruitment practices between sites and during the course of the outbreak and the availability of treatments and facilities (e.g., ICUs and ventilators) across the range of resource settings. ISARIC’s collaborative approach to research outputs addresses these challenges by involving all staff who contributed to the collection of data in the review of the analysis plans and manuscripts. When designing an analysis plan, researchers must also consider which data are and are not available from each site and account for high levels of missingness, particularly during regional peaks in COVID-19 transmission. The CDISC SDTM data model was selected for harmonisation of these data, specifically because it captures these aspects of data providence. Those using the dataset benefit from the richness of the model; however, they will need to master the challenges of its complexity. Tools to support understanding of the data model can be found at https://www.iddo.org/tools-and-resources/data-tools.

### Collaborative research

The ISARIC WHO characterization protocol has proven to be a successful strategy for generating standardized data from multiple sites that international researchers can access for analysis^18,21,22,24–27^. Having a pre-prepared protocol for clinical investigation of an emerging infectious disease established before the beginning of the COVID-19 pandemic allowed us to gather patient data very early in the pandemic. As a result, contributors benefited from clinical data captured in other regions before they experienced cases and improved confidence in a larger dataset. By implementing systems to harmonize global data, ISARIC and IDDO have made international collaboration more efficient^1^. The evolution of these systems, including integrating epidemiological and genomic data to address new types of research questions, is in progress. Finally, ISARIC’s data governance model allows members and non-members to propose research questions that could be answered using this dataset, which has helped advance science and empowers scientists worldwide^4,10,20^. This open and collaborative approach maximizes the scientific utility and public health impact of global data. With a focus on ensuring the representation of patient data and researchers from lower-resourced settings, the ISARIC network has accelerated understanding of COVID-19, advanced preparedness for future pandemics, and raised the bar on global collaboration for health.

## Supplementary information

Supplementary Table 1

## Data Availability

Processing codes for the ISARIC COVID-19 database are openly available online, and contributions from the research community to share these codes are encouraged. For this reason, a public code repository has been created along with this manuscript to develop and share code collectively: https://github.com/ISARICDataPlatform/ISARICBasics.git. The content of this repository is under continuous development. Still, it has been seeded with code to generate patient-level datasets suitable for statistics and machine learning research, such as patient demographic, comorbid conditions at the time of admission, application of treatments, and severity scores, among others. It is possible for the research community to directly submit updates, improvements, and additions to the repository via GitHub. Moreover, a Jupyter Notebook containing the code used to generate the tables and descriptive statistics included in this paper is openly available on GitHub.
