# Supplementary material for: ISARIC-COVID-19 dataset: A Prospective, Standardized, Global Dataset of Patients Hospitalized with COVID-19
Source: Sci Data. 2022 Jul 30;9:454. doi: 10.1038/s41597-022-01534-9 (PMC9339000; doi:10.1038/s41597-022-01534-9)
Supplement: Supplementary file 1 — Supplementary Table 1 [file 41597_2022_1534_MOESM1_ESM.docx]

**Supplementary Table 1.** An overview of the data tables comprising the ISARIC COVID-19 database.

| **Domain** | **Domain Name** | **Number of Variables** | **Rows** | **Unique patients with data reported** | **Description** |
| --- | --- | --- | --- | --- | --- |
| **IE** | Inclusion/Exclusion Criteria | 12 | 847,889 | 285,959 | Individual-level fulfilment of inclusion/exclusion criteria |
| **DM** | Demographics | 15 | 708,231 | 708,158 | Essential standard, non-clinical variables that describe an individual |
| **SC** | Subject Characteristics | 9 | 5,589 | 1,864 | Non-clinical variables that describe an individual |
| **ER** | Environmental Risk | 14 | 1,254,087 | 675,537 | Assessments of potential exposures to or risk factors associated with the disease |
| **SV** | Subject Visits | 5 | 295,314 | 295,275 | Start and end date of hospitalization and visits |
| **HO** | Healthcare Encounters | 23 | 2,041,390 | 704,229 | Inpatient and outpatient healthcare events. |
| **RP** | Reproductive System Findings | 16 | 277,050 | 273,678 | Pregnancy-related variables |
| **PO** | Pregnancy Outcomes | 9 | 2,710 | 1,355 | The outcome of pregnancy; pre-term, live birth |
| **VS** | Vital Signs | 20 | 4,024,655 | 294,911 | Measurements of the body's essential functions are monitored during visits or hospitalization. |
| **SA** | Clinical and Adverse Events | 21 | 31,923,533 | 677,926 | Clinical events of interest |
| **LB** | Laboratory Results | 21 | 8,717,839 | 253,641 | Laboratory test data (except microbiology) |
| **MB** | Microbiology Specimen | 25 | 1,629,843 | 647,568 | Detection, identification, and quantification of microorganisms |
| **IN** | Treatments and Interventions | 34 | 32,368,574 | 684,313 | Experimental, concomitant, and prior medications and treatments |
| **RS** | Disease Response and Clinical Classification | 19 | 1,003,741 | 257,137 | Variables assess disease response to therapy or record clinical classification based on published criteria. |
| **DS** | Disposition | 13 | 695,096 | 670,202 | Medical status or outcomes |
| **CQ** | COVID-19 Follow-Up Questionnaire | 17 | 566,309 | 18,878 | Standardized COVID-19 questionnaires |
| **TS** | Trial Summary | 11 | 246 | Study level domain | Variables that describe the trial |
| **TI** | Trial Inclusion Exclusion Criteria | 5 | 44 | Study level domain | Trial-level inclusion and exclusion criteria |
| **DI** | Device Identifiers | 6 | 79 | Study level domain | Parameters to distinguish among devices. |
